# Probabilistic modeling of the semantic fluency task with extended Markov networks

**DOI:** 10.3758/s13428-026-03048-3

**Published:** 2026-05-22

**Authors:** Miguel López, Natividad Hernández Muñoz, Carmela Tomé Cornejo

**Affiliations:** 1 Inst. Investigación en Ingeniería, University Miguel Hernandez, Elche, Spain; 2 Departamento de Lengua Española, University of Salamanca, Salamanca, Spain

**Keywords:** Fluency tasks, Markov modeling, Lexical retrieval, Semantic networks

## Abstract

Semantic verbal fluency tasks (SFT) provide a window into the structure and dynamics of the mental lexicon by eliciting word sequences guided primarily by semantic associations. We propose a probabilistic framework that models SFT as censored random walks on semantic networks, extended with pseudo-nodes to account for local and global jumps. This representation enables the integration of associative retrieval and sudden resets, capturing both clustering and switching processes. To assess model quality, we define a suite of complementary metrics—global likelihood, frequency likelihood, and bigram relevance—that evaluate not only overall fit but also the distributional properties of word associations. Using a dataset of 677 lists in the category “clothing,” we benchmark existing techniques against our proposed BIGRAM-CN model, which combines statistical constraints with empirical frequencies of word-to-word transitions. Results show that BIGRAM-CN avoids overfitting, generalizes across training and test data, and synthesizes realistic lists more accurately than prior approaches. This work advances computational models of lexical retrieval and offers practical tools for comparing populations, categories, and cognitive profiles in both linguistic and psychological research.

## Introduction

Throughout life, experience provides us with information about our understanding of the world, much of which we encode in the meaning of the words that form the language we use to communicate as social beings. Studying the conceptual structure of a language offers insight into how the mind organizes our perception of reality. The information that shapes the meaning of linguistic units derives not only from our interaction with the environment, but also from the linguistic context in which these words are used. All this lexical-semantic knowledge has been studied through the theoretical construct of the mental lexicon (Pirrelli et al., 2020). Conceived as a dynamic cognitive system that integrates word forms, meanings, and their interrelationships, it underlies both conscious and unconscious lexical activity (Jarema & Libben, 2007). As such, it constitutes a foundational element for both linguistic theory and psycholinguistics. Consequently, the study of mental lexicon representation has been a central topic in cognitive sciences.

From a cognitive psychology perspective, understanding how the cognitive system represents, organizes, and accesses a vast and constantly evolving body of verbal knowledge is essential for explaining the general principles that govern human thought. Characterizing the mental lexicon is crucial not only for knowing how language is processed, acquired, and degraded, but also for understanding cognition in a broader sense—for instance, in relation to knowledge representation, the general mechanisms of semantic memory, or cognitive performance (Kumar, 2021; Zemla, 2022).

From a linguistic standpoint, reflecting on how speakers manage lexical knowledge, construct word meanings, and relate these meanings within the linguistic system and in actual language use is essential across various disciplines and theoretical paradigms (Marconi, 2000; Meara, 1996). Furthermore, this reflection sheds light on the categorizing processes that also determine the nature of the language used to communicate and to create a socially shared world (Kleiber, 1990). In particular, understanding how lexical competence develops is fundamental for language teaching and learning (Nation, 2022). Learning a word is an associative process that involves integrating the new item into the existing lexical structure, thereby establishing connections with previously acquired words and giving rise to an autonomous lexical network in the second language (Borodkin et al., 2016).

In this context, the development of precise models for investigating the mental lexicon has wide-ranging implications. Crucially, a model that can both represent the structure of the lexicon and simulate its navigation during retrieval—in addition to accounting for the dynamism that characterizes lexical activity—provides an essential bridge between experimental data and theoretical models (both linguistic and psycholinguistic), enabling consideration of the speaker’s role as an active agent in linguistic activity.

One enduring and influential perspective on the architecture and dynamics of the mental lexicon conceptualizes it as a metaphorical network, in which each word is represented as a node and the relationships between words as connecting edges (Aitchison, 2008; Meara, 2009). This view aligns with early network activation theories, according to which word retrieval is driven by the spread of activation from a cue toward semantically connected concepts (Collins & Loftus, 1975).

In contrast, spatial models posit that words occupy positions within a multidimensional semantic space, where distances correspond to degrees of similarity. These distances were initially derived from human similarity judgments but more recently have been estimated from patterns of word co-occurrence in large text corpora. Computational frameworks such as HAL (hyperspace analogue to language; Burgess & Lund, 1997) or LSA (latent semantic analysis; Landauer & Dumais, 1997) embody this vector-based, distributed view of meaning, in which semantic similarity emerges from statistical regularities in language use (see Günther et al., 2019, for a review). More recently, both approaches have served as the basis for the development of formal models of lexical search and retrieval, as reviewed later.

Semantic networks are conceived as metaphorical and abstract simulations of knowledge organization, grounded in the connections that exist between words. Although—like most mental models—this kind of representation is not directly observable, it can still be empirically investigated through psycholinguistic data, offering insight into the storage, structure, and retrieval of knowledge through lexical units (Beckage & Colunga, 2016).

While not all semantic networks are derived from experimental or psychological data—for instance, those generated from computer-simulated dictionaries or lexicographic tools (Steyvers & Tenenbaum, 2005), or those based on co-occurrence patterns in textual corpora (De Deyne et al., 2016)—networks constructed from data elicited through speaker performance in specific linguistic tasks provide a more ecologically valid perspective on knowledge representation. Consequently, accurately estimating semantic representations from experimental data has become a key computational challenge in contemporary psychology (Zemla, 2022).

Among the psycholinguistic tasks most commonly used to construct semantic networks, *semantic fluency tasks*—also referred to as *category fluency* or *exemplar generation* in psychology, and *lexical availability* in linguistics—stand out (Mazzuca & Majid, 2023; Zemla, 2022). In these tasks, participants are asked to produce a list of words related to a given prompt (e.g., a semantic category) within a limited time frame (typically 1–3 min) or up to a fixed number of exemplars. Retrieval processes in these tasks are semantically guided by the stimulus or category (e.g., “animals,” leading to “dog, cat, horse”), and participants typically produce semantically related words in sequence through associative retrieval mechanisms. Researchers have found that typicality, familiarity, and age of acquisition of words affect semantic fluency: items more typical, more familiar, and learned earlier tend to appear more frequently and in higher positions in the lists (Fatima et al., 2021; Hernández Muñoz et al., 2006).

Semantic fluency tasks have a long-standing tradition in both psychology and linguistics. They have been extensively employed in psychological research since the early twentieth century (Battig & Montague, 1969; Bousfield & Sedgewick, 1944; Thurstone, 1938), as well as in linguistic studies (Michéa, 1953). One of their key advantages is their ability to delimit specific lexical fields guided by categorization processes, which are known to be central to the cognitive organization of knowledge and to the structuring of both experience and lexical-semantic levels (Hernández Muñoz et al., 2025).

From the speakers’ competence, performance on category fluency depends on the integrity of semantic memory organization and the efficiency of search processes and lexical retrieval. These components involve distinct but interrelated cognitive demands that operate jointly and require precise timing and efficient extraction of lexical material, as well as attentional, cognitive control, and executive processes (Chertkow & Bub, 1990; Crowe, 1998; Dorchies et al., 2024). Within this set of processes, vocabulary size appears to interact with executive control mechanisms (Bose et al., 2022). Godefroy et al. (2023) proposed a model of semantic fluency involving three main types of processes operating together: linguistic processes, a general attentional activation process, and a strategic search process (see Dorchies et al., 2024, for a review).

To the extent that performance on this task provides information about the integrity of semantic memory and the coordinated functioning of the aforementioned processes, semantic fluency has been widely used not only as a tool for basic research but also as an instrument for the assessment and diagnosis of cognitive decline in diverse clinical populations (Avery & Jones, 2018; Neergaard et al., 2025; Zemla & Austerweil, 2017). In order to capitalize on the informational value of these data beyond the mere number of items produced and their order of recall, Troyer et al. (1997) introduced the measures of clustering and switching. These metrics made it possible to characterize lexical retrieval patterns which consist of the production of sequences of semantically related words (clusters) and shifts between conceptual subcategories (switches). However, subsequent research has highlighted important theoretical and methodological limitations of these measures. In particular, the delineation of clusters relies on subjective judgments, raising concerns about reliability and validity, and both measures show a strong dependence on the total length of the list (Avery & Jones, 2018; Borodkin et al., 2016; Neergaard et al., 2025).

These limitations have motivated the development of computational models aimed at formalizing human behavior in the semantic fluency task. Within this framework, one of the most influential proposals is that of Hills et al. (2012), who introduced an analogy between memory search and optimal foraging strategies observed in animal behavior. The model proposed by Hills et al. (2012) posits that semantic memory is organized into patches, and that speakers dynamically alternate between local exploitation of a patch (guided by a semantic similarity cue) and global exploration of the network (guided by a word-frequency cue) in order to locate a new resource patch once the current one has been depleted. The optimal moment to make these transitions is predicted by the marginal value theorem, which states that a forager should leave a patch when the local rate of return falls below the average rate of return across the entire system.

In contrast to the cue-switching model, the random walk model proposes that the jumps observed in lexical production do not necessarily reflect an optimization strategy (see Avery & Jones, 2018, for an explicit comparison between the two models and an insightful discussion of methodological confounds in the literature). Assuming a network-based representational structure, the original model by Abbott et al. (2015) implements a censored random walk (CRW)—a sequence of transitions in which the next node is selected randomly among the neighbors of the current node; previously visited nodes are traversed but not included in the sequence—that locally traverses the network as a function of its relational structure, where a jump allows a return to the starting node or category, from which local exploration is resumed. From this perspective, patterns consistent with optimal foraging emerge as an epiphenomenal property of network structure rather than as the outcome of an explicit cognitive search strategy. In a subsequent development, Zemla and Austerweil (2017) argue that a CRW with a priming component offers the best compromise for capturing the frequency and clustering effects observed in semantic fluency data. In this proposal, the jump is conceived as a transition that improves the model’s fit to behavioral data, without necessarily implying a “reset” to the root node or requiring the process to be optimal. Unlike earlier conceptions, jumps involve a flexible relocation that reflects plausible search mechanisms capable of reproducing empirical regularities.

In this vein, the present study aims to develop and validate a more fine-grained modeling of the semantic fluency task, aimed at delineating differentiated speaker profiles at both the individual and population levels (for example, between men and women, native and non-native speakers, or healthy and clinical populations), in line with recent proposals by Fatima et al. (2021) or Neergaard et al. (2025). Building on the framework proposed by Zemla and Austerweil (2017, 2018), we introduce a series of modifications aimed at overcoming certain limitations of existing models that undermine their reliability. These limitations include a reliance on binary networks (Zemla & Austerweil, 2018), which may not accurately represent empirically observed asymmetries in transition frequencies (Avery & Jones, 2018); an excess of parameters not justified by the complexity of the task, leading to overfitting and poor generalization to novel data (Jun et al., 2015); and a dependence on external data or manual classifications, such as taxonomic norms (Avery & Jones, 2018), which constrains objectivity and automation. Finally, most current models lack generative capacity, meaning they cannot produce synthetic lists that are statistically equivalent to human performance.

Our proposal addresses these challenges and succeeds in improving the model’s discriminative capacity, reducing overfitting, and endowing it with generative capabilities. Although the semantic fluency task is modeled as a CRW, the resulting strategy is consistent with Hills’ optimal foraging theory, viewing retrieval as a balance between exploiting local semantic patches and exploring new ones. In our model, clustering and switching behavior are explicitly and coherently represented through two complementary mechanisms: statistically significant links between words for local movement (clustering) and transitions to a specialized jump node for global resets (switching). The censoring process is considered an active process of executive inhibition that prevents the repetition of previously produced items. Rather than “burning bridges,” nodes already visited act as cognitively economical “semantic bridges” that facilitate continued exploration. This interpretation allows the model to realistically capture how search processes traverse and revisit existing patches, leveraging primed concepts to reach new neighbors. This framework allows us to model, visualize, and analyze both aspects of the retrieval process within a single, unified structure.

Moreover, the goal of using the model to characterize divergent production patterns leads us to define a set of specific metrics that address lexical productivity, response selection, and frequency, as well as grouping behavior. This approach allows, on the one hand, an assessment of whether the lists generated by an individual or a population are implausible with respect to the model, and, on the other hand, the identification of the underlying cause of such discrepancies. In this way, the model exhibits high potential for application across diverse fields such as psycholinguistics, applied linguistics, sociolinguistics, and clinical neuropsychology, while also contributing to the theoretical debate surrounding optimal foraging versus random walk models of semantic memory search.

Thus, the present work makes the following key contributions:

- A unified generative model: Building on Abbott’s framework, we propose a weighted transition network that incorporates a specialized jump node. This architecture allows for independent modeling of clustering and switching probabilities based on the current location within the network. Unlike traditional models, our approach maintains a full Markovian structure, which not only enables the generation of realistic synthetic data but also allows for the application of advanced Markovian analysis techniques to the retrieval process.
- Optimized model fitting: We introduce the BIGRAM-CN estimation technique, which maximizes likelihood over training data while significantly reducing the number of parameters. By using the conceptual network (CN; Goñi et al. 2011) as a structural prior, the model avoids overfitting; this is empirically demonstrated by achieving statistically equivalent likelihoods on test datasets. Furthermore, the synthetic lists generated by this model preserve the core statistical properties of the original human data.
- Objective evaluation metrics: We establish a suite of metrics designed to evaluate models without relying on subjective external taxonomies. These metrics assess global likelihood but also verify whether the synthetic output maintains the same word frequency patterns and associative structures as human speakers. This provides a benchmark for comparing different modeling approaches against a common, objective standard of performance.

The remainder of this paper is organized as follows: Section "Theoretical framework and reference models" details the theoretical and mathematical framework, describing the most relevant concepts of Markov processes and reviewing the baseline models used for reference. Section "Materials and methods" provides a description of the data and the models proposed in this work, as well as their application in generating synthetic lists. In Section "Model evaluation metrics", we outline the evaluation protocol for all models, demonstrate how they can be visualized, and describe the parameter optimization process. Section "Results" presents the outcomes for both the reference and the proposed models, along with a concise description of the most significant findings. Section "Discussion" examines the cognitive implications of these results, particularly regarding semantic memory access and the advantages of the “jump node” mechanism in modeling switching and clustering behavior. Finally, Section "Conclusions" summarizes the main contributions of this work, highlights the model's superior generalization capacity and its potential applications in clinical diagnostics, and suggests directions for future research.

## Theoretical framework and reference models

The process of word list generation in semantic fluency tasks (SFT) can be represented as a CRW through a semantic network, modeled as a Markov process. In this framework, each word corresponds to a node in the network, and transitions between words are governed by probabilistic rules derived from empirical data.

### Theoretical concepts of Markovian processes

A Markov process is defined by a transition probability matrix (TPM) *P*. Each element *p*_*i,j*_ expresses the probability of going from node *i* to node *j*. The matrix *P* is generally constructed from an adjacency matrix *A*, in which each element *a*_*i,j*_ represents the strength of the link between nodes *i* and *j*. The process to obtain *P* from *A* is described as

1 $${p}_{ij}=\frac{{a}_{ij}}{{\sum}_{k}{a}_{ik}}$$

Adjacency matrices are called unweighted if the values allowed are 0 and 1, representing the existence of a given edge. Additionally, they are called undirected if the weight between two nodes is the same in both directions (the matrix is, in this case, symmetrical). However, TPMs will not generally inherit these qualities, in the sense that if a node *i* and a node *j* are connected to two and three different nodes, respectively, the probability of going from *i* to *j* will be 50%, while the inverse journey will have a probability of 33%. The method for determining the elements of the adjacency matrix is one of the key aspects of an SFT model.

As was mentioned in the introduction, it is common in the SFT to produce sequences of semantically related words followed by changes between subcategories. The use of Markov processes allows the modeling of this behavior by the introduction of a pseudo-node accessible from every other node and from which any other node is reachable. In this way, a jump can be included in an adjacency matrix (*A*) to form a new adjacency matrix (*A′*) by assigning a vector of weights $\widetilde{\theta }$ in which each element $\widetilde{{\theta}_{i}}$ represents the strength of the link between node *i* and the reset node. Additionally, a vector of landing weights $\widetilde{{\rho}_{j}}$ is required, representing the probability of landing on node *j*. The way that each weight on both vectors is determined depends on the model used. Equation 1 can be applied to *A′* to obtain a TPM that includes a jumping node.

Moreover, jumps can also be introduced directly to a TPM by defining a vector of jumping probabilities $\theta$, in which each element ${\theta}_{i}$ represents the probability of jumping from node *i*, and a vector of landing probabilities $\rho$, in which each element ${\rho}_{j}$ represents the probability of landing on node *j*. Note that each row of *P* must be normalized by a factor of $\left(1-{\theta}_{i}\right)$ to ensure that *P*_*j*_ remains row-stochastic. This process is described by Eq. 2 for a constant jump probability and a landing probability distribution based on frequency, although other methods can be used.

2 $$\begin{array}{c}Pj=\left[\begin{array}{cc}0& \rho \\ \theta & {p{\prime}}_{i,j}\end{array}\right]\\ {\rho}_{j}=\frac{{f}_{j}}{\sum {f}_{j}}; {\theta}_{i}=cte.;{p{\prime}}_{i,j}=(1-{\theta}_{i})\cdot {p}_{i,j}\end{array}$$

**Fig. 1:**
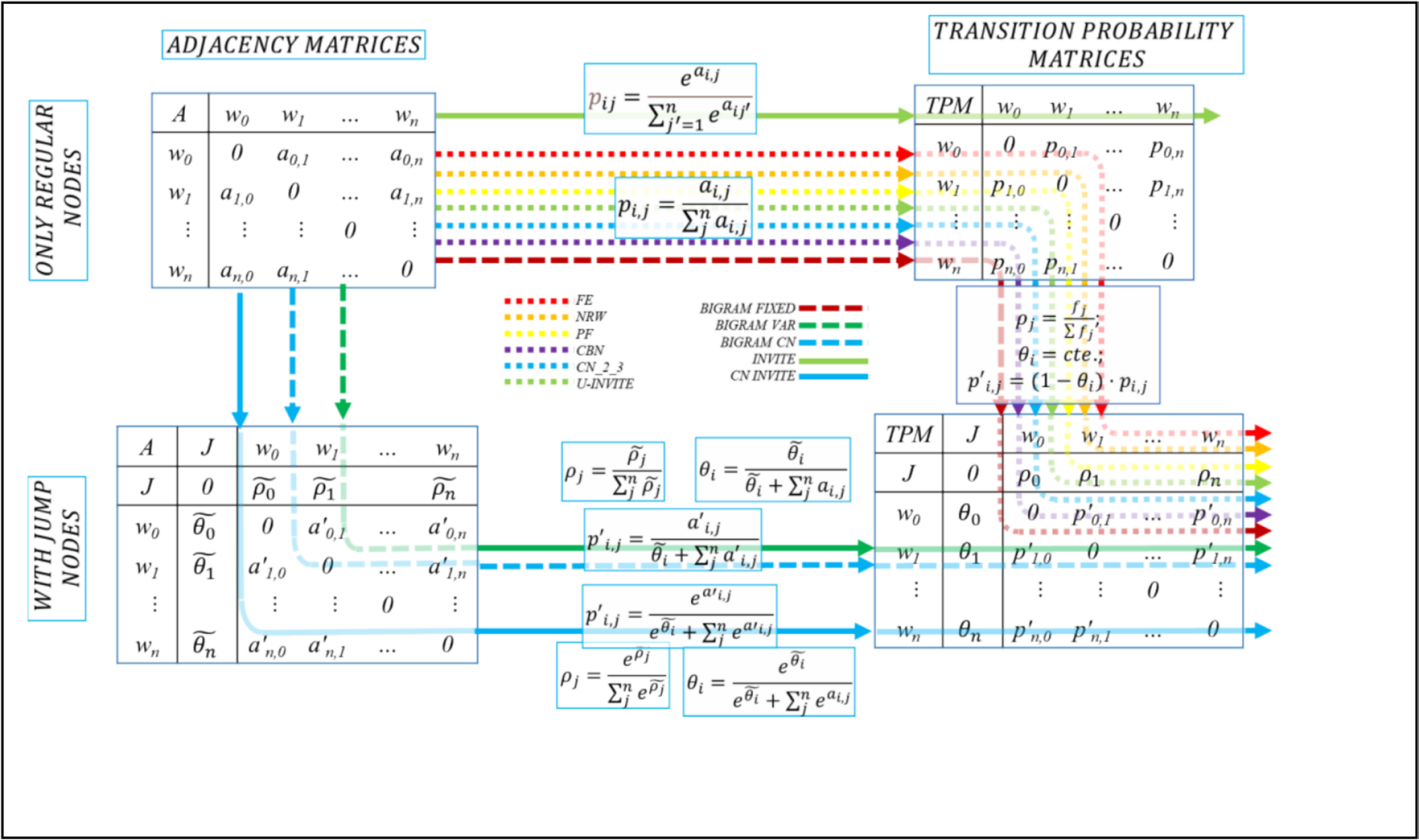
Schematic view of the construction of each model’s TPM from the initial and method-specific adjacency matrix or from the beta parameter matrix for INVITE-based models

The paths followed by each of the models analyzed is shown in Fig. 1, where the transitions from adjacency to TPMs are detailed. The process of adding jumps on TPMs is also included because it is the same for all methods that use it. However, the technique used to include jumps in the adjacency matrix is not described on the schematic, as it is method-specific.

Likelihood serves as the fundamental metric for assessing model goodness of fit by quantifying the probability that the observed data were generated by the model; in the context of Markov processes, it is formally defined as the product of the transition probabilities between successive states. The likelihood function can be written as shown in Equation 1, as proposed in Jun et al. (2015) and Zemla and Austerweil (2018). Note that the TPM *P*_*j*_ can include jump nodes which are always censored by considering them as previously visited nodes:

3 $${\mathbb{P}}\left({L}^{1},{L}^{2},...,{L}^{N}|{P}_{J}\right)=\prod_{i=1}^{N}\left({\mathbb{P}}\left({w}_{i}^{1}\right)\prod_{j=1}^{{\lambda}_{i}-1}{\mathbb{P}}\left({w}_{i}^{j+1}|{w}_{i}^{1:j}\right)\right)$$

Perseverations are avoided by censoring, that is, the output sequence is restricted to first-visit transitions; any subsequent visits to previously activated nodes are censored (omitted) from the chain. This censoring process carries a fundamental implication: it transforms the static network into a path-dependent system where effective transition probabilities evolve with every word spoken. By treating previously visited nodes as “transient,” the model accounts for the probability mass flowing not only through direct connections but also through paths of already visited states. This mechanism allows for effective transitions between nonadjacent nodes when the path between them has already been explored. Consequently, the probability of reaching a jump node is no longer a fixed property of the current state, but a dynamic outcome of the trajectory; as a semantic neighborhood is depleted, the relative likelihood of an exit jump increases, reflecting the cognitive reality of local resource exhaustion during the fluency task.

While the inclusion of an explicit jump node offers significant advantages for modeling specific behaviors—such as quantifying cluster exhaustion or isolating clustering and switching dynamics—it can introduce artifacts in other types of analysis. Specifically, in a matrix with an explicit jump state, the stationary distribution can be distorted by the probability mass assigned to the jump node itself. Furthermore, calculating mean first-passage times becomes problematic, as any transition through the jump node is counted as a two-step process.

To resolve this issue, the jump node can be mathematically reabsorbed to produce an equivalent transition matrix without the explicit jump state (${P}_{ABS}$). This reabsorption process, detailed in Eq. 4, preserves the effective transition probabilities between semantic nodes while eliminating the structural bias of the jump node. This dual representation allows for the modeling of switch-and-cluster dynamics while maintaining a clean, interpretable framework for global network analysis.

4 $${P}_{ABS}= P{\prime}+\theta \rho$$

### Reference models

#### Unweighted

The unweighted methods, in general, will not contain a link corresponding to any possible transition found in a list. Therefore, in order to assess the likelihood of lists from the training set but also from the test set, jumps must be added. In Abbott et al. (2015), Zemla and Austerweil (2017), and Zemla et al. (2020), this is done outside the Markov process as a post-process, in which a constant probability of jump is added for each transition. However, the formulation proposed here (see Fig. 1) is mathematically equivalent and it provides the advantage of maintaining the Markovian nature, making it possible to apply all the analytical tools available for such processes.

Each method describes a different way to implement an adjacency matrix (*A*). A TPM (*P*) is then obtained by applying Eq. 1. The final TPM with jumps (*Pj*) is obtained using Eq. 2, as detailed in Fig. 1. The landing distribution ($\rho$) used is proportional to the frequency of each word (*fi*). The value for the jumping probability ($\theta$) that maximizes likelihood is found using a fivefold validation process with the training data.

- First edge (FE) (Abrahao et al., 2013; Jun et al., 2015; Zemla & Austerweil, 2018): The adjacency matrix is inferred by adding an edge between the first and second words in every list. This technique discards most of the information from the list, assuming that the first pair of words tend to have a strong relation. This avoids the presence of false edges on the network.
- Naïve random walk (NRW) (Jun et al., 2015; Lerner et al., 2009; Zemla & Austerweil, 2018): This technique is similar to FE, except that the whole list is used to add edges. It assumes that every list is constructed by a random walk, and an edge is added for every pair of adjacent words on the lists. The likelihood of this network on the training data is nonzero, since every transition in the list is possible, but any transition that is not present on the training lists would yield a zero likelihood until jumps are added. In contrast to the FE method, false edges will be added to the network whenever two unrelated words are mentioned together on any list.
- PathFinder (PF) (Paulsen et al., 1996; Zemla & Austerweil, 2018): The PF method calculates a proximity matrix based on the distance between pairs of words in all lists following Chan et al. (1995). This matrix is then used to obtain an unweighted, undirected network following the Pathfinder method of Schvaneveldt (1990). The PF method uses two parameters to control the sparsity of the resulting network, these parameters are set so that the sparsest network is inferred as implemented in Zemla and Austerweil (2018).
- Conceptual network (CN) (Goñi et al., 2011; Zemla & Austerweil, 2018): This method uses statistical analysis to determine whether the frequency with which a pair of words appears within a given distance across the lists can be expected from chance. It is described in detail in Goñi et al. (2011); however, a brief overview is provided in Appendix 1 for reference, as it is relevant for the development of the proposed model.
- U-INVITE (UI) (Zemla & Austerweil, 2018): This method is a derivation of the one described in Jun et al. (2015), which generates weighted networks and will be reviewed in the following section. In both cases, the methods assume that a fluency list is generated by a CRW, and they attempt to estimate the network that maximizes the likelihood of the data, as defined in Eq. 3, that originate a given set of fluency lists. The U-INVITE method uses a stochastic search to find the unweighted semantic network that maximizes likelihood. The first step is to obtain a nonzero likelihood network, which can be obtained using some of the already described techniques. From such network, an iterative process is applied which toggles edges of the network (edges are either pruned or added to the network). If the changed network has a higher likelihood, then the change is accepted. The process is applied until there are no more possible changes that increase the likelihood. The heuristics for choosing the edges in a particular order are comprehensively described in Zemla and Austerweil (2018).
- Correlation-based network (CBN) (Kenett et al., 2013): This method estimates semantic networks based on the co-occurrences of words on the same lists regardless of their position. Given *N* lists with *W* unique tokens, an *N* × *W* binary matrix (*F*) is built by assigning the element *F*_*i,j*_ the value of 1 if the token *j* appears on list *i*. The Pearson correlation coefficient is calculated for every pair of words, and each pair is sorted from highest coefficient to lowest. The network is built by adding an edge to the network from the sorted list until the network is no longer planar (Tumminello et al., 2005).

#### Weighted methods

Weighted methods produce networks with weighted edges. Larger weights represent stronger links between the words and ultimately a higher transition probability. The use of weighted networks was questioned by Zemla and Austerweil (2018), as it is suggested that the binary underlying structure is sufficiently informative and that obtaining weights for the edges adds too many degrees of freedom to the problem. However, Avery and Jones (2018) noted that random walks perform better on weighted networks. The following methods deal with these drawbacks in different ways.

The definition of a model generally starts by estimating the weights in the adjacency matrix. From there, as shown in Fig. 1, the process can go directly to estimating a TPM without jumps (INVITE) or a TPM with added jumps, which are equally probable (BIGRAM-FIXED). The possibility of obtaining an adjacency matrix with jumps which in turn becomes a TPM with node-specific jumping probability shown in Fig. 1 is explored in the proposed methods (BIGRAM-VAR, BIGRAM-CN, and INVITE-CN).

- BIGRAM-FIXED: The concept of bigrams in fluency tasks is well established (Feng & Liu, 2023). When two words appear consecutively in a list, they form a bigram. The BIGRAM-FIXED method adds an edge for each new bigram found in the fluency lists (similarly to the NRW method) and assigns the total number of occurrences of that bigram across all lists as the edge weight, forming the adjacency matrix. The TPM is obtained applying the same process used for the unweighted methods.
- INVITE (Jun et al., 2015): The article introduces a new probabilistic model called INitial-VIsiT Emitting random walk (INVITE) to model human memory search in verbal fluency tasks. The model does not generate a typical adjacency matrix, but rather a free-parameter matrix ${\boldsymbol{\beta}}$ from which a TPM is obtained using Eq. 6. This is so that the restrictions on *P* (*P* must be row-stochastic) are removed from the optimization problem. From this matrix, it is possible to compute the model’s likelihood on a set of word lists. The model is initialized with a random matrix and uses an optimization algorithm (averaged stochastic gradient descent) to maximize the likelihood. The cost function used is

5 $$\underset{\upbeta }{\mathrm{min}}-{\sum}_{i=1}^{N}{\sum}_{j=1}^{{\lambda}_{i}-1}\mathrm{log}{\mathbb{P}}\left(\left.{w}_{i}^{j+1}\right|{w}_{i}^{1:j};{\boldsymbol{\beta}}\right)+\frac{1}{2}{C}_{\beta }{\sum}_{i\ne j}{\beta}_{ij}^{2}$$

6 $${P}_{ij}=\frac{{exp}^{{\upbeta}_{ij}}}{{\sum}_{{j}{\prime}=1}^{n}{exp}^{{\upbeta}_{i{j}{\prime}}}},\hspace{1em}\forall i,j.$$

where *N* is the number of lists, ${\lambda}_{i}$ is the length of list *i*, ${w}_{i}^{j}$ is the *j-th* word on the *i-th* list, and *C*_*β*_ is an L_2_ regularization that functions as a smoothing mechanism rather than a sparsification tool. Because parameters are mapped to a probability space, penalizing high absolute values prevents the emergence of overconfident distributions. Instead, the optimizer favors uniform transition probabilities in the absence of empirical evidence. This is designed to prevent the model from overfitting to noise in low-frequency semantic transitions. The details of the mathematical justification for this process can be found in Jun et al. (2015).

## Materials and methods

The following subsections describe the data used in this research and the details of each proposed method for modeling the SFT.

### Data

The full dataset contains 680 lists comprising a total of 449 different words (types). Participants were preuniversity students (17 to 18 years old), 290 male and 390 female, and all native speakers of the Spanish spoken in central Spain. They completed a semantic fluency task during a class period in their educational institutions. The task adopts the methodological parameters from lexical availability studies in linguistics, as part of the Pan-Hispanic Lexical Availability Project (López Morales, 2014) and obtained the approval of the Research Ethics Committee of the University of Salamanca. The assessment instrument consisted of a paper-based questionnaire including columns for the completion of 16 semantic categories. Participants were allotted 2 min to generate responses for each category. All responses were subsequently transcribed into a digital database and systematically reviewed to eliminate errors and duplicate entries. Inflectional variants of the same lexical item were standardized under a single lemma (camisa and camisas in camisa/s “shirt/s”). Forms in which variation in gender or number entailed a semantic or conceptual distinction were treated as separate entries (gorro “hat” and gorra “cap”). It should be noted that the data were collected via written fluency, which may differ from spoken fluency in timing, dynamics, and the types of words elicited (Hernández Muñoz, 2010; Tomé Cornejo, 2023). Mandera et al. (2017) demonstrated that models trained on spoken-language registers, specifically film and television subtitles, exhibit different sensitivity from models trained on written corpora. Therefore, the present results should be interpreted with caution regarding their direct extrapolation to spoken contexts. The category chosen for this study was “ropa” (clothing items). Three lists were removed because they contained at least 10 words considered off-topic by the research team. After this filtering, the dataset consists of 677 lists with 396 different words (0.24 idiosyncratic words per list).

One of the main issues when estimating models from data is overfitting. To identify this problem, 20% of the lists (135) were set aside to evaluate whether the developed models fit both the training and the test data. Similarly, for those models requiring parameter tuning, a fivefold cross-validation technique was used to validate the parameter optimization.

The testing data contain 46 words that were not present in the training data (44 were idiosyncratic terms, each appearing in the list of a different participant). In order to assess the likelihood of the testing data, these words were swapped with words from the training dataset with equal frequency.

### Proposed methods

The following methods are designed to address the limitations identified in the reference benchmarks, namely the inability of unweighted networks to distinguish between stronger and weaker links, the risk of overfitting in weighted networks, and the constraint of a constant jumping probability. The CN method is used in some models as a mask to sparsify the matrix. The preference for this method is based on the performance of the CN unweighted.

- **CN variations (unweighted):** In this section, we propose exploring the influence of network construction parameters based on word category. The original CN model, as described in Goñi et al. (2011), specifies fixed parameter values, but we consider it relevant to explore alternative configurations. To this end, tests will be conducted with different settings for the minimum word distance (*d*_*min*_) required to define a co-occurrence and the minimum number of co-occurrences needed to establish a link (*f*_*min*_). The parameter *d*_*min*_ can be interpreted cognitively as the semantic activation span or the buffer size during the retrieval process, while *f*_*min*_ serves to distinguish between idiosyncratic associations and the robust semantic consensus of the community. These variations are a deliberate approach to model specific processes underlying the fluency task, like the span of semantic activation or the threshold of shared community consensus.
- **BIGRAM-IND (weighted):** Starting with the adjacency matrix from the BIGRAM-FIXED model, it is common practice to prune edges with weights below a certain threshold; however, this threshold is scale-dependent and does not have a fixed or optimized value. Typically, edges with a weight of 1 are removed (Hernández Muñoz et al., 2025). The threshold used in this research was found using the fivefold cross validation system, and for our case it has a value of 4. The weights of the edges removed are pooled together and assigned to the edge linking the node *i* and the jump state. In this way, every node *i* has different individual jumping probability (${\theta}_{i}$).
- **BIGRAM-CN:** This method combines unweighted networks with the BIGRAM method for weighted networks. It first uses a binary adjacency matrix (*A*_*CN*_) obtained by the CN unweighted method to determine which edges are relevant. Then the weight of the edges is calculated using the BIGRAM method, providing the weighted adjacency matrix ${{A}_{B}}^{\gamma }$, in which each weight ${a}_{{B}_{i,j}}^{\gamma}$ corresponds to the number of times words *i* and *j* appear together on the training lists raised to an exponent $\gamma$, a scaling parameter that controls the sensitivity of the model to co-occurrence counts. A $\gamma$ value of zero generates a binary matrix, while higher exponents favor larger differences between links. The final weighted matrix (*A*_*BCN*_) is obtained by the element-wise multiplication of *A*_*CN*_ and *A*_*B*_. Additionally, the method provides a way to estimate the probability of jumping from each node. The weight of the edges from each node to the jump state corresponds to the sum of the weights of the edges deemed irrelevant. The calculations for the matrix *A*_*BCN*_ and the weight vector for jumps ($\widetilde{\theta }$) are detailed in Eqs. 7 and 8.

7 $${A}_{BCN}={A}_{CN}\odot{{A}_{B}}^{\gamma},$$

8 $$\widetilde{\theta }=\left(\left(1-{A}_{CN}\right) \odot {A}_{BCN}\right){1}_{W},$$

where $\widetilde{\theta }$ is the vector of jumping weights ($\widetilde{{\theta}_{i}}$ is the weight from node *i* to the jump node), $\odot$ represents the Hadamard product, and $1$ and ${1}_{W}$ are a squared matrix and a column vector of 1s.

- **INVITE-CN:** This method applies the principle of the CN model variations to the INVITE technique. One of the drawbacks of the INVITE method is that it is prone to overfitting. However, if the parameters over which the optimization is implemented are limited to those identified as relevant, then overfitting could be prevented. This method applies the same optimization algorithm as INVITE but uses a binary matrix obtained using the CN method to identify the fixed and the optimizable parameters. The jump probability vector ($\theta$) and the landing distribution ($\rho$) are also included in the optimization.

### Creation of synthetic lists

A key advantage of our modeling approach is its generative capability, which allows for the simulation of synthetic word lists to determine whether the model can replicate human-like production patterns. To this end, synthetic lists were generated with lengths sampled from a discretized normal distribution fitted to the empirical training data. For all models featuring a jump node, the generation process was initialized at that state; for the INVITE model, the starting word was instead selected following a distribution proportional to observed word frequencies. In all cases, a first-visit censoring rule was applied, recording nodes only upon their initial activation. Finally, synthetic datasets of the same size as the training set were generated to compare the models’ performance across several metrics.

As shown in Fig. 1, all methods except INVITE include a jump node in the TPM that can be revisited at any step of the random walk. Therefore, synthetic lists include jumps following the probability defined in the TPM and the trajectory of the walk.

## Model evaluation metrics

In this section, we describe the different metrics used to evaluate the performance of each model. While consistent likelihood between training and test sets is used to rule out overfitting, we argue that this metric alone is insufficient to guarantee model validity. Models with statistically equivalent likelihoods can still produce anomalous sequences that fail to replicate human patterns of word frequency, word order (Fatima et al., 2021), or associative transitions. Therefore, we introduce a set of complementary metrics applied across all datasets to assess whether the synthetic output remains behaviorally grounded. By integrating these measures, we provide a comprehensive comparison to identify the model that best captures the underlying dynamics of the fluency task.

### Quality metrics

The global likelihood is the probability of the observed data under the current model parameters. It serves as a key measure of how well the model explains the data. In addition, it was considered relevant to assess whether the synthetic lists generated by the models obtain scores similar to those of real lists on two criteria, which can also be used to identify anomalies in lists produced by individual subjects or groups. List length, which is a common measure of SFT performance, is also analyzed to show that it can be modeled by a discretized normal distribution, which in turn is used to determine the length of the synthetic lists. The proposed criteria aim to answer the following questions:

- Which words? The number of words is not the only meaningful indicator, as the specific words included in the list also convey important information. Examining whether an individual, a group, or a model tends to produce overly rare or overly common words can provide insight into their lexical access and cognitive strategy.
- How do the words associate among them? The order in which words are produced is also meaningful, as it is common for certain words to elicit others. Analyzing these associations can help assess whether an individual, group, or model follows expected patterns of semantic organization.

To ensure the model's generalizability, it will be verified that the training and test data yield similar scores in terms of likelihood and each of the defined criteria. This ensures that the model not only fits the training data well but also accurately evaluates new, previously unseen data. In our experiments, 80% of the data will be used to train and validate the models, and the remaining 20% will be used to assess overfitting.

- **Global likelihood (*gLL*):** The concept of likelihood was defined in Section “**Theoretical concepts of Markovian processes**”, and the formulation is shown in Eq. 3. However, in practice, it is common to use the negative log-likelihood (NLL) instead of the likelihood because it improves numerical stability (by converting a product of small probabilities into a sum of log-probabilities, avoiding underflow), simplifies computation (since sums are easier to handle than products), and aligns with standard optimization methods, which typically minimize a function rather than maximize it. Since the logarithm is monotonically increasing, minimizing NLL is equivalent to maximizing likelihood. The actual reported value for the *gLL* in this paper is the average probability for each transition, i.e., the geometric mean of the probability of all transitions, as shown in Eq. 9. This number is more intuitive, as it represents the probability of predicting the next word correctly.

9 $$gLL={\left(\prod_{i=1}^{N}\left({\mathbb{P}}\left({w}_{i}^{1}\right)\prod_{j=1}^{{\lambda}_{i}-1}{\mathbb{P}}\left({w}_{i}^{j+1}|{w}_{i}^{1:j}\right)\right)\right)}^{{~}^{1}\!\left/ \!{~}_{\sum {\lambda}_{j}}\right.}$$

The key to calculating transition probabilities in CRW is provided in Jun et al. (2015) and Zemla and Austerweil (2018). This is a complex problem because there are infinite routes to the next node when one can go through already visited nodes, including going back and forth an infinite number of times. Each transition is considered an independent absorbing walk over a set of nodes (the ones on the list). Previously traversed nodes are treated as transient, while the rest of the nodes are absorbing. Therefore, the probability of transitioning from node *k* to node *k+*1 is equal to the probability of a walk starting on node *k* and being absorbed by node *k+*1 where previously traversed nodes (*1* through *k*) are transient and the rest are absorbing. The details for these calculations are explained in the references above. The addition of the JUMP state does not conflict with this procedure, as JUMP is always the starting node and is always considered transient.

We apply each evaluation method to the training, testing, and synthetic datasets. For each combination, we report the *gLL* with 95% confidence intervals (calculated via bootstrapping) and the Bayesian information criterion (BIC) for model comparison. To characterize the distribution of these likelihoods, we verify that the data follow a beta distribution using Kolmogorov–Smirnov (KS) tests and Bayes factors (BF_01_). For these fits, we provide the $\alpha$ and $\beta$ parameters, the mode, and the shortest credible interval (SCI). Finally, we compare the testing and synthetic results against the training baseline to check for statistical equivalence. This comparison is performed using the KS test, the BF_01_, and the distributional overlap, which measures the percentage of similarity between the generative output and the training data.

- **Word frequency likelihood (*fLL*)**: This measure assesses whether the words in a single list or a group of lists are as frequent as expected based on the training data. While standard approaches often compare group-level means or empirical distributions of word frequency, our modeling approach requires a metric capable of assessing the generative plausibility of individual lists. The word frequency distribution derived from the training set is used to compute the likelihood of each list. The likelihood of a list is calculated as the geometric mean of the probabilities of the individual words, which ensures comparability across lists of different lengths. The frequency likelihood formula is described in Eq. 10:

10 $${fLL}^{j}={\left(\prod_{i=1}^{{\lambda}_{j}}1-{\left(1-\frac{{f}_{{w}_{i}}}{N}\right)}^{{~}^{{\lambda}_{j}}\!\left/ \!{~}_{\overline{\lambda }}\right.}\right)}^{{~}^{1}\!\left/ \!{~}_{{\lambda}_{j}}\right.}$$

where ${fLL}^{j}$ is the frequency likelihood of the *j*-th list, *f*_*wi*_ is the number of lists that contain the word *w*_*i*_, *N* is the total number of lists, *λ*_*j*_ is the length of list *j*, and $\overline{\lambda }$ is the average list length. For a set of lists, the reported value is the geometric mean of the *fLL* for all lists.

To provide a comprehensive evaluation of this metric, we report a multilayered set of metrics. First, we establish the aggregate performance by calculating the mean *fLL* for each dataset, accompanied by 95% confidence intervals derived via bootstrapping (1,000 iterations). Second, we characterize the structural properties of the likelihood distributions. We assess the fit of an empirical beta distribution to the *fLL* values, reporting the estimated shape parameters $\alpha$ and $\beta$, the mode, and the 50% SCI to pinpoint the highest-density region of the data. The validity of this beta assumption is strictly tested using both frequentist (*p*-values) and Bayesian frameworks (BF_01_), where the latter quantifies the evidence in favor of the distributional fit. Finally, we evaluate the consistency across datasets (training, test, and synthetic). We employ the KS test and its corresponding BF_01_ to determine whether the samples originate from the same underlying distribution. This is further corroborated by the distributional overlap, providing an intuitive percentage-based measure of similarity between the training baseline and the generative output.

- **Normalized number of relevant bigrams (nRB):** This measure assesses whether a given list has the expected number of common associations for its number of words. This is conceptually related to measuring cluster switches. However, traditional “switching” metrics rely on external categorization taxonomies (e.g., predefined subcategories of animals) which can be subjective and language-dependent. In contrast, our nRB metric is fully data-driven and unsupervised. The first step for obtaining this measure is to determine which pairs of words are significatively related. To this end, the CN method (Goñi et al., 2011) is used. When the relevant word couples are identified, the number of instances in which any of these couples appear on each list is determined, as described in Eq. 11.

11 $$C\left({L}_{l}\right)=\sum_{i=1}^{{\lambda}_{i}-1}1\left(\left({w}_{i},{w}_{i+1}\right)\in R\right)$$

where *C*(*L*_*j*_) is the count for list *j*, ${\lambda}_{i}$ is the number of words in *L*_*j*_, ***1***(*·*) is the indicator function that equals 1 if the condition inside the parentheses is satisfied and 0 otherwise, *w*_*i*_ is the *i*-th word in list *j,* and *R* is the set of relevant word pairs.

The count *C* is highly correlated with the number of words in the list. To eliminate this correlation, we will use the derived measure C*, as described in Eq. 12.

12 $${C}^{\ast}\left({L}_{j}\right)=C\left({L}_{j}\right)-m\cdot \left({\lambda}_{j}- \overline{\lambda }\right)$$

where *λ*_*j*_ is the length of list *j,* $\overline{\lambda }$ is the average list length, and *m* is the slope obtained by fitting a line to the points $\left({\lambda}_{j},C\left({L}_{j}\right)\right)$.

The analysis of relevant bigram counts follows a structured approach focused on three dimensions: distributional shape, statistical equivalence, and lexical alignment. First, given the apparent Gaussian nature of the data, we describe each dataset using the mean and standard deviation. The assumption of normality is verified through *p*-values and Bayes factors (BF_01_). Second, we assess how closely the test or synthetic datasets mirror the training baseline. We report the results of the KS test and its associated BF_01_ to check for statistical identity. This is complemented by the distributional overlap, which provides a direct percentage of the similarity between the distributions. Finally, to determine whether the models capture the actual content of human associations, we calculate the coefficient of determination (*R*^2^) between the bigram frequency vectors. While the previous metrics evaluate the global count of bigrams, this *R*^2^ analysis quantifies the specific alignment between the bigrams generated and those observed in the training data, ensuring the model replicates the precise relational structure seen on the training data.

### Visualizations of the network

**Fig. 2:**
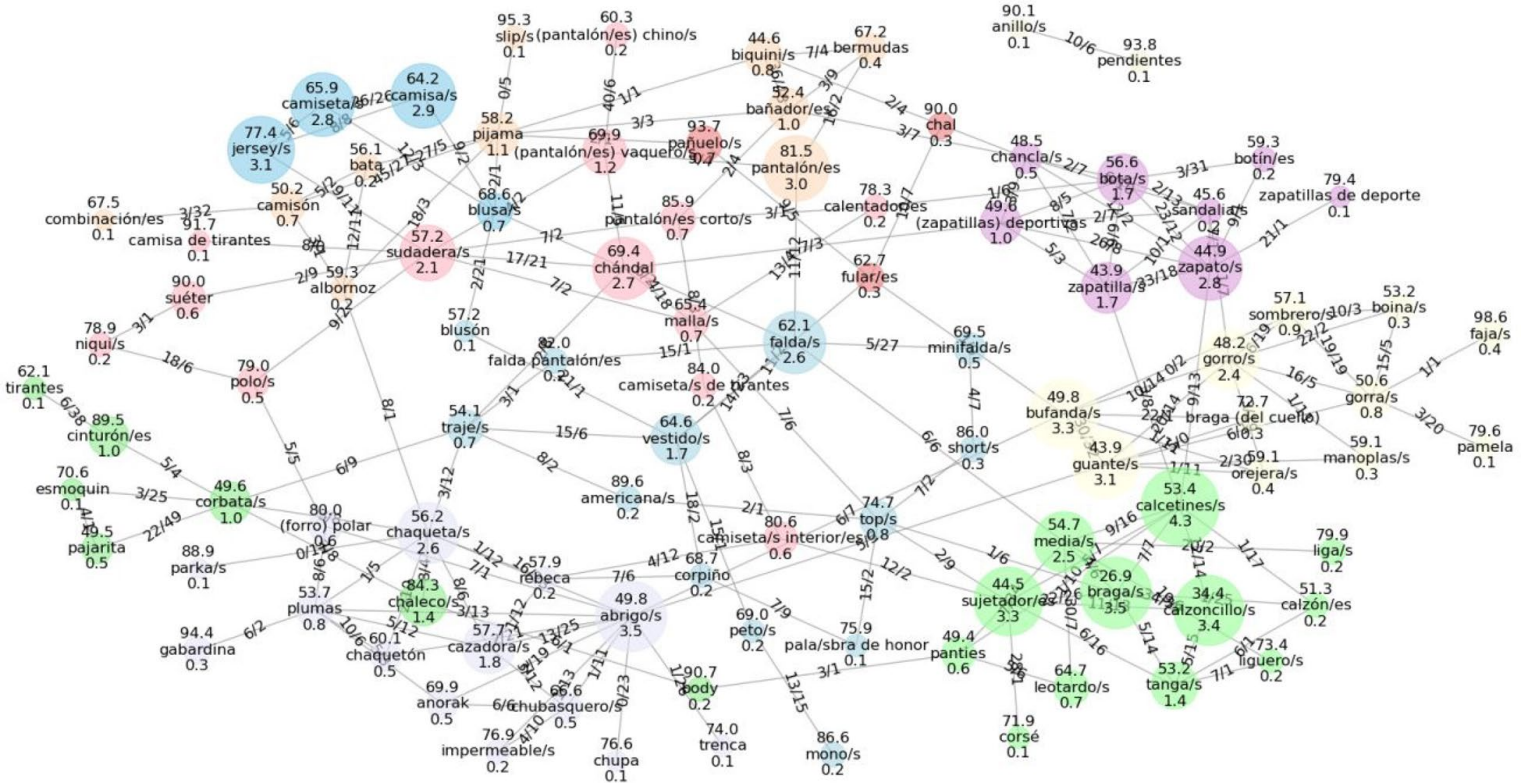
Graph representing the local component (isolated nodes are excluded) of the transition probability matrix obtained using the CN-BIGRAM method. On the nodes, the number above represents the jumping probability from the node, and the number below is the probability of landing on the node after a jump. On the edges, the label (A/B) represents as A the probability of going from the first node to the second, while B represents the inverse path. The first and second nodes are assigned alphabetically

The ability to visualize the network is valuable both for facilitating human comprehension and for providing accurate intuitions about its underlying mechanisms. However, large networks with high numbers of nodes and edges are hard to visualize. Fortunately, the structure of the TPM with jumps allows us to separate the network into two components: a local component that includes all direct transitions between nodes, and a global component that includes the transitions going through a jump state. The global component offers very little visual information, as it can be summarized by the jumping and landing distributions. In fact, 87.7% of the transitions in our training dataset were between nodes present in the local component shown, although they might have happened through the jump node. Therefore, the local component allows us to visualize the probability of the vast majority of transitions. Figure 2 shows a representation of the CN network obtained.

### Optimization of the model parameters

**Fig. 3:**
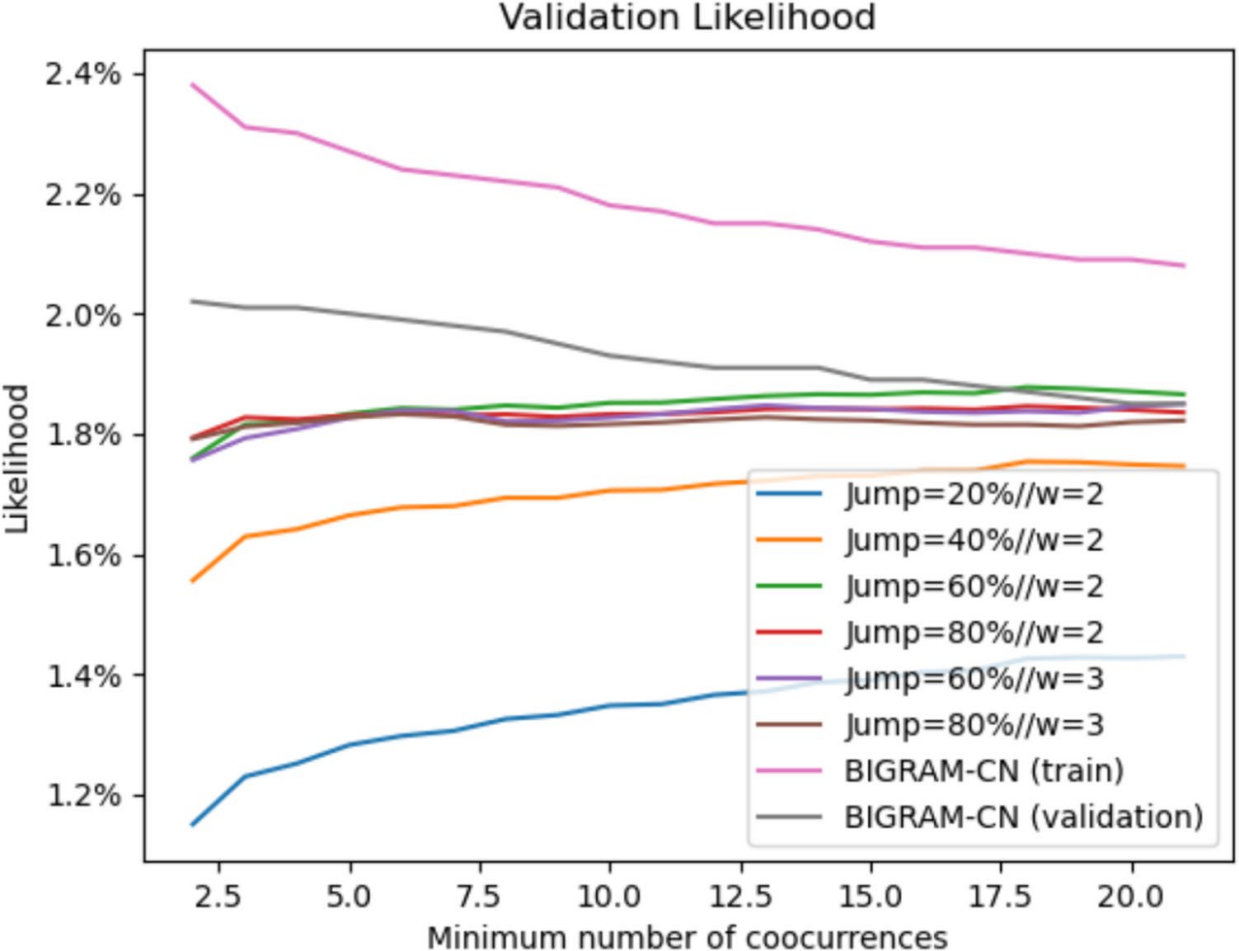
Likelihood of validation data using different parameters in the unweighted CN model. The increase in the minimum number of co-occurrences causes an increase in the likelihood due to the pruning of unlikely transitions. The unweighted feature forces the model to remove these edges, while weighted methods simply assign them a lower weight

To determine the optimal parameters for the models, we performed a fivefold cross-validation procedure in which each fold contained complete lists, ensuring no overlap across training and validation sets. The analysis revealed a clear optimal value for the jump probability for each method and for the co-occurrence window size for the CN method. However, the co-occurrence frequency threshold showed no clear optimum: increasing the threshold consistently improved the model's likelihood on validation data, as shown in Fig. 3. This behavior is explained by the uniform distribution of probability across the binary transitions: including low-frequency links dilutes the probability mass and reduces the likelihood of high-frequency transitions. These findings highlight a limitation of the binary, unweighted approach, motivating a transition to models with weighted connections that can more accurately reflect the strength of associations between words. Therefore, the frequency threshold for the unweighted and weighted application of the CN approach is optimized using the BIGRAM-CN method following the same fivefold cross-validation method. Our results indicate that increasing the co-occurrence threshold led to a consistent decrease in likelihood for both training and validation sets. This suggests that retaining low-frequency associations is vital for capturing the richness of human associative patterns. However, while the lowest possible threshold preserves the most information, we discarded a threshold of 2 because it showed clear signs of overfitting.

## Results

The results for the different models are presented in this section. First, the training and testing data are analyzed using the measures described in Section "Model evaluation metrics". Then, each of the methodologies is tested to obtain a likelihood value and a set of measures to assess whether the list generated by the model matches the training and testing data.

### Measuring the training and testing data

Before evaluating model performance, we assessed whether the training and test sets exhibit similar distributions across the defined measures. This step ensures that the criteria are stable and meaningful, and that the training data are representative of the test data. Furthermore, these results establish the benchmark that synthetic lists produced by each model are expected to match.

**Fig. 4:**
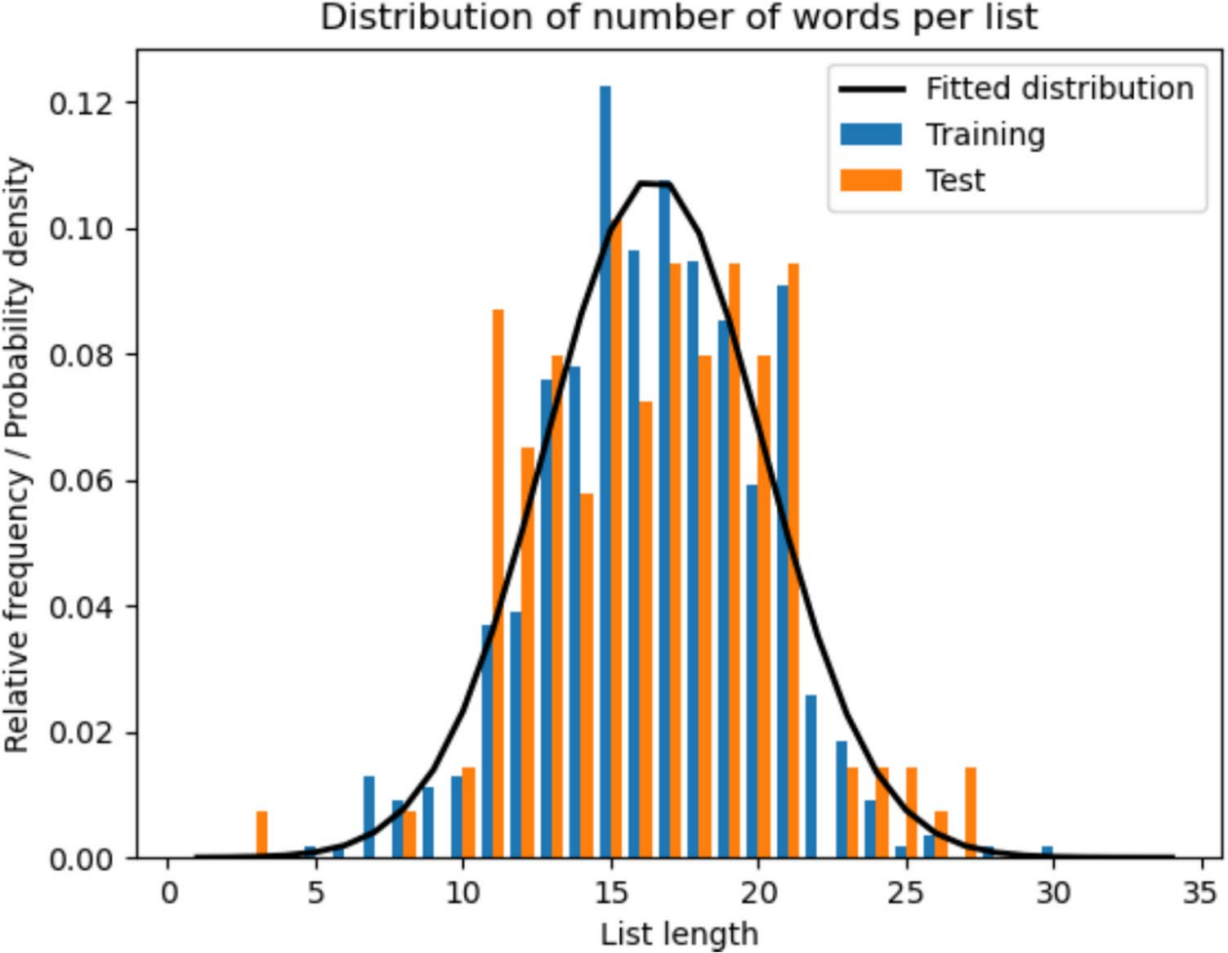
Histogram of the list lengths of the training and testing datasets. The distribution fitted to the training data is also shown

- **List length:** Table 1 presents the mean and standard deviation of the discretized normal distribution fitted to the training data. The *p*-values obtained from the KS test, tailored for this specific distribution, indicate that the null hypothesis—that both datasets follow the fitted distribution—cannot be rejected. Additionally, the BF_01_ Bayes factors provide strong to moderate evidence that the list follows the fitted distribution. For visual reference, Fig. 4 shows both datasets with the fitted distribution.

**Table 1:** Number of words on training and testing lists

Average list length	Standard deviation	*p*-value (training)	BF_01_ (training)	*p*-value (testing)	BF_01_ (testing)
16.47	3.69	0.706	**12.35**	0.589	**8.55**

These results confirm that the list lengths follow a discretized normal distribution. This finding was used to determine the length of the synthetic lists generated by each model; therefore, it is unnecessary to verify that the generated lists follow this distribution, as it is enforced by design. Nevertheless, including this analysis was important to validate the use of this distribution as a reasonable way to set list lengths, even though it does not model the internal cognitive processes that lead individuals to end the task.

- **Word frequency likelihood:** The *fLL* results were calculated for the training and testing data. Additionally, and to provide context, they were calculated for lists that selected words randomly from a uniform distribution and from a distribution proportional to frequency. Table 2 summarizes these results.

**Table 2:** *fLL* on training, testing, and synthetic lists using uniform and frequency-based distributions

Dataset	fLL % (95% CI)	*p*-value_beta (BF_01_)	Alpha	Beta	Low-25% %	Mode %	Hi-25% %	*p*-value_train (BF_01_)	Overlap %
**Training**	21.2 (20.5–21.9)	**0.088** **(13.9)**	5.7	19.7	11.7	20.1	30.2	DNA	DNA
**Testing**	19.6 (17.9–21.1)	**0.201** **(6.4)**	4.0	14.6	11.6	19.5	29.6	**0.423** **(4.05)**	**90.1**
**Uniform**	0.9 (0.8–0.9)	**0.072** **(12.7)**	4.0	427.8	0.4	0.7	1.3	0.0 (0.0)	0.0
**Frequency**	17.8 (17.2–18.3)	**0.468** **(8.7)**	6.7	29.7	10.9	17.0	24.5	0.0 (0.0)	77.6

**Fig. 5:**
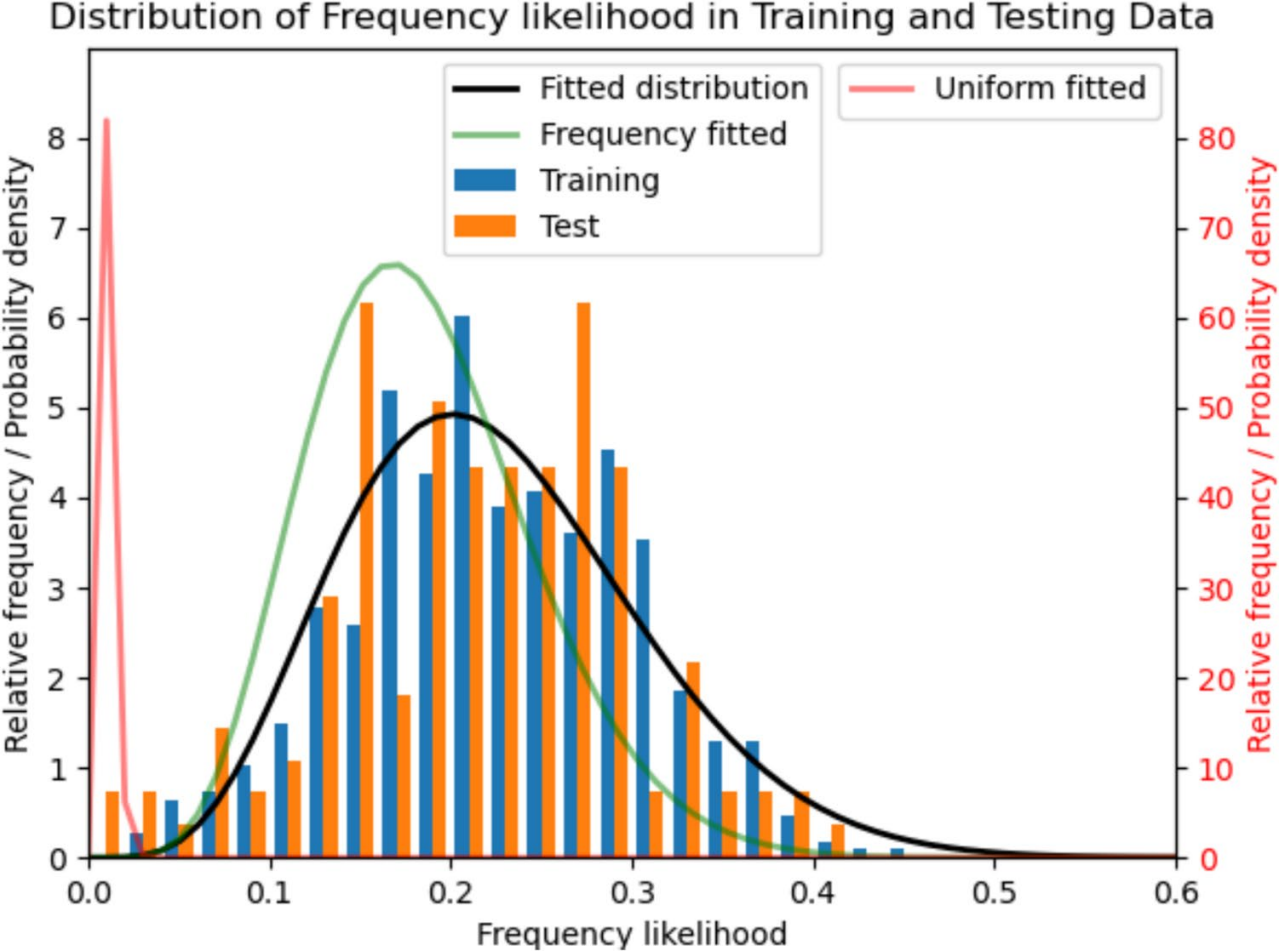
Histogram of the frequency likelihood of the training and testing datasets. The distribution fitted to the training data is also shown. For reference, the distributions fitted to synthetic lists obtained using a word-frequency and uniform distribution is also shown

The results demonstrate a clear statistical equivalence between the training and testing datasets. The *fLL* for the training and test sets show overlapping confidence intervals, suggesting that the model's performance remains stable across different samples. This similarity is further supported by the KS test, which yielded a nonsignificant *p*-value and a BF_01_ providing strong evidence that both datasets originate from the same underlying distribution. A key finding is the difference in variability. The confidence intervals for the *uniform* and *frequency* synthetic models are notably narrower than those of the human datasets. This indicates that real-world data are subject to a higher degree of variance, likely driven by behavioral factors like clustering, that these simpler generative models do not replicate. Finally, the fit of the beta distribution is validated across all conditions. The BF_01_ values, coupled with nonsignificant *p*-values, provide robust evidence that the beta distribution is the appropriate model for describing the *fLL* density in all analyzed groups. The list generated by choosing from a distribution proportional to frequency has a slightly lower likelihood than the training and testing lists. This can be explained by the censoring step in the generation process. Since words can only appear once in a list, the actual frequency of highly mentioned words is underestimated in the distribution, leading to an overrepresentation of less probable words. These results are further illustrated in Fig. 5.

**Fig. 6:**
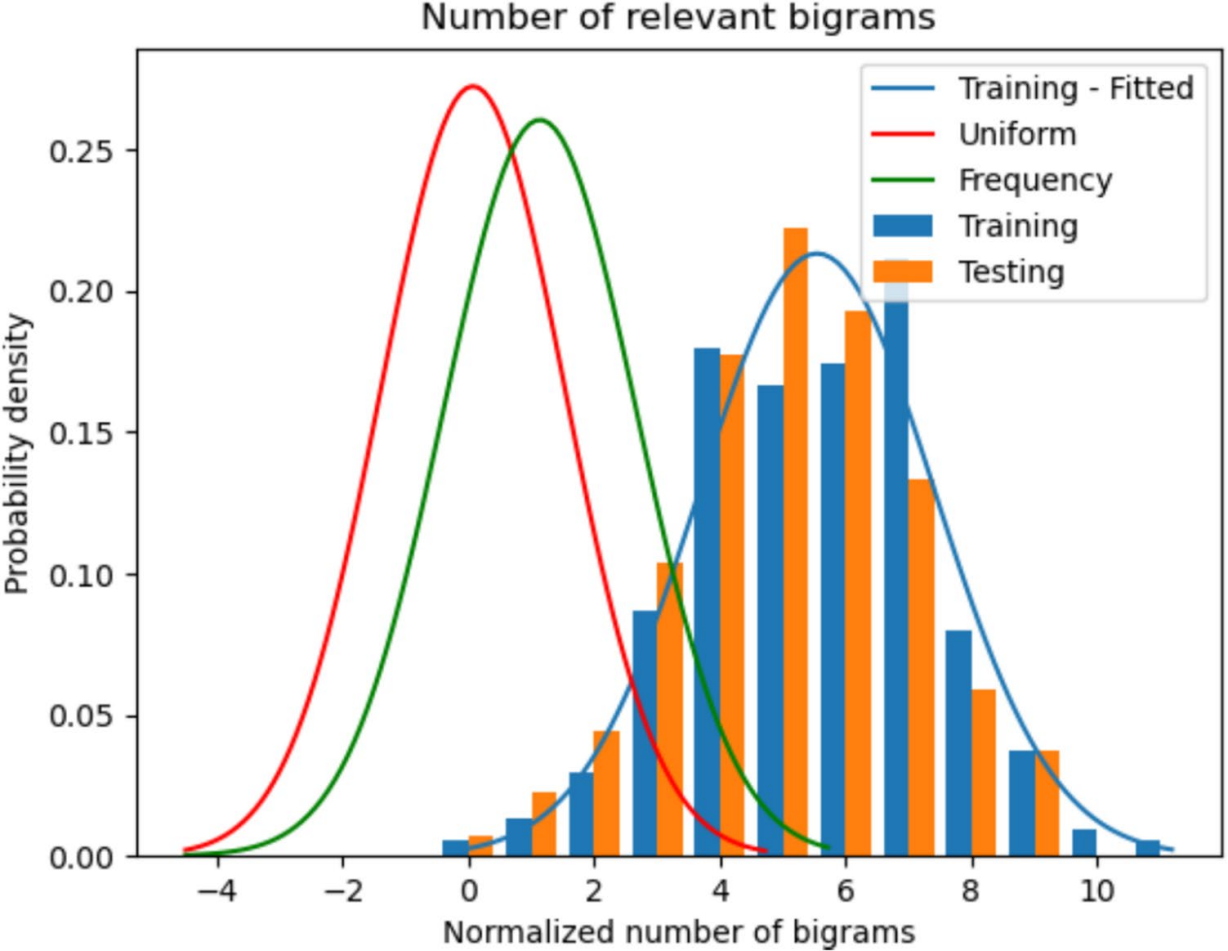
Histogram of the normalized relevant bigrams of the training and testing datasets. The distribution fitted to the training data is also shown. For reference, the distributions fitted to synthetic lists obtained using a word-frequency and uniform distribution is also shown

- **Number of relevant bigrams (nRB):** The results for the training and testing data along with the two sets of synthetic lists are presented in Table 3 and also visualized in Fig. 6.

**Table 3:** nRB on training, testing, and synthetic lists using uniform and frequency-based distributions

Dataset	Mean	Standard deviation	*p*-value -Gaussian-(BF_01_)	*p*-value -training-(BF_01_)	Overlap %	***R***^**2**^
**Training**	5.37	1.87	**0.173** **(13.5)**	DNA	DNA	DNA
**Testing**	5.07	1.78	**0.987** **(6.99)**	**0.090** **(2.1)**	**93.1**	0.86
**Uniform**	0.04	1.35	**0.210** **(13.5)**	0.000 (0.0)	9.7	−0.42
**Frequency**	1.10	1.49	**0.447** **(14.3)**	0.000 (0.0)	20.3	−0.04

The results for the relevant bigram counts (nRB) highlight a sharp contrast between human performance and the simplified synthetic models. The training and testing datasets exhibit nearly identical profiles in terms of mean and standard deviation, both of which are significantly superior to the other groups. As anticipated, the uniform distribution produces virtually zero bigrams, while the frequency distribution generates a slightly higher count simply because it favors common words, more likely to be in the set of relevant bigrams. Statistical validation confirms that all datasets follow a Gaussian distribution, supported by robust Bayes factors and nonsignificant *p*-values. Crucially, the test set is the only one that demonstrates genuine equivalence to the training data, evidenced by a high distributional overlap and a supportive BF_01_. This consistency is further reinforced by the *R*^2^ score, which confirms that the specific lexical associations produced by the testing group closely match those established by the training group.

### Assessment of unweighted methods

- **Global likelihood:** The global likelihood measure for the unweighted methods is reported in Table 4. Each method was implemented with a jumping probability that optimized global likelihood on the validation data. All evaluated models produce *gLL* distributions that fit a beta distribution, as confirmed by nonsignificant *p*-values and supportive Bayes factors BF_01_. A defining characteristic of most methods is the requirement for a high fixed jump probability, typically 0.8. This high probability shifts the structural relevance from local node-to-node links toward the global landing distribution. The only exception is the U-INVITE method, which utilizes a lower jump probability (0.4). This suggests that the UI model may rely on spurious links to fit the training data, attempting to mimic global behavior through local connections that do not necessarily reflect the underlying structure of the test data. This hypothesis is supported by the larger BIC (each possible link is a parameter for this model) and the poor performance on the test dataset.

**Table 4:** Global likelihood results of unweighted methods

Model	Data	JP	*gLL* % (95% CI)	BIC	*p*_value beta dist (BF_01_)	Alpha	Beta	Bottom-25%	Mode	Hi-25%	*p*_value train (BF_01_)	Overlap
**FE**	Train	0.8	1.52 (1.46–1.58)	74,364	**0.233** **(4.9)**	6.0	351.1	0.75	1.42	2.23	NA	NA
	Test		1.34 (1.22–1.46)	19,651	**0.501** **(6.4)**	3.7	232.0	0.47	1.17	2.16	0.195 (3.2)	87
	Synt.		1.47 (1.43–1.51)	74,964	**0.365** **(13.5)**	8.3	528.5	0.87	1.36	2.04	0.000 (0.0)	89
**NRW**	Train	0.8	**1.99** **(1.96–2.01)**	69,587	**0.292** **(13.0)**	36.5	1,766.1	1.59	1.97	2.35	NA	NA
	Test		1.38 (1.26–1.50)	19,521	**0.056** **(6.2)**	4.3	268.8	0.54	1.23	2.14	0.000 (0.0)	47
	Synt.		1.21 (1.19–1.24)	78,380	**0.596** **(12.8)**	12.3	964.9	0.78	1.16	1.58	0.000 (0.0)	26
**PF**	Train	0.8	1.85 (1.80–1.90)	70,858	**0.349** **(13.0)**	10.3	519.5	1.18	1.77	2.52	NA	NA
	Test		1.41 (1.29–1.53)	19,447	**0.164** **(7.3)**	4.3	261.0	0.60	1.25	2.23	0.000 (0.0)	72
	Synt.		1.34 (1.30–1.37)	76,655	**0.774** **(12.3)**	9.5	665.1	0.84	1.27	1.85	0.000 (0.0)	61
**CBN**	Train	0.8	1.78 (1.72–1.83)	71,593	**0.657** **(14.7)**	6.5	329.9	0.92	1.66	2.56	NA	NA
	Test		1.50 (1.36–1.65)	19,150	**0.131** **(5.5)**	3.8	211.8	0.53	1.31	2.39	0.048 (0.4)	84
	Synt.		1.44 (1.40–1.48)	75,322	**0.824** **(14.5)**	7.9	510.4	0.86	1.33	2.04	0.000 (0.0)	74
**UI**	Train	0.4	1.83 (1.80–1.86)	1,125,111	**0.315** **(10.5)**	21.2	1,112.6	1.35	1.78	2.26	NA	NA
	Test		1.25 (1.16–1.35)	916,346	**0.343** **(3.1)**	5.3	371.5	0.57	1.14	1.87	0.000 (0.0)	55
	Synt.		1.38 (1.35–1.42)	1,130,092	**0.459** **(14.3)**	9.0	611.0	0.81	1.29	1.87	0.000 (0.0)	60
**CN_** **2_3**	Train	0.6	**1.97** **(1.89–2.06)**	**69,735**	**0.111** **(8.5)**	4.5	199.1	0.81	1.75	3.00	NA	NA
	Test		**1.71** **(1.54–1.88)**	**18,576**	**0.359** **(7.4)**	3.4	166.5	0.59	1.45	2.80	**0.080** **(1.8)**	90
	Synt.		**2.05** **(1.99–2.12)**	**69,051**	**0.998** **(13.9)**	6.9	307.6	1.12	1.89	2.92	**0.012** **(12.5)**	89

While both FE and CN show consistent behavior between training and test sets, the CN model performs better across all metrics. It achieves a higher test *gLL* (1.71%) and the lowest BIC, indicating a superior balance of fit and simplicity. Furthermore, the CN model shows a stronger overlap (90%) with the training baseline, and its synthetic lists are the only ones statistically compatible with the original human data (BF_01_ = 12.5).

- **Word frequency likelihood:** The word frequency likelihood measure is reported in Table 5. The examination of the data reveals that all generative methods produce *fLL* distributions that follow a beta model, as confirmed by the corresponding *p*-values and Bayes factors. However, none of these methods successfully replicates the training distribution, with all yielding a *p*-value of 0.000 when compared to the training baseline. Generally, these models do not perform better than a simple selection based on global frequencies, which previously showed an *fLL* of 17.8% and an overlap of 77.6%. The results suggest that introducing unweighted direct transitions between related words distorts the natural frequency of the words within the lists. Methods that generate sparse matrices, such as FE and CN_2_3, achieve the highest *fLL* values at 19.0% and 17.9%, respectively. In contrast, the UI method, which prioritizes local links with a lower jump probability (40%), results in a significantly lower likelihood of 8.6%.

**Table 5:** Frequency likelihood results of unweighted methods

Dataset (jump prob.)	fLL % (95% CI)	*p*-value_beta (BF_01_)	Alpha	Beta	Low-25% %	Mode %	Hi-25% %	*p*-value_train (BF_01_)	Overlap %
**Training**	21.2 (20.5–21.9)	**0.088** **(13.9)**	5.7	19.7	11.7	20.1	30.2	NA	NA
**Testing**	19.6 (17.9–21.1)	**0.201** **(6.4)**	4.0	14.6	8.9	17.9	29.7	**0.386** **(4.0)**	**90**
**FE (0.8)**	19.0 (18.4–19.5)	**0.630** **(14.7)**	6.6	27.5	10.7	17.5	25.8	0.000 (0.0)	84
**NRW (0.8)**	15.6 (15.1–16.0)	**0.395** **(13.9)**	6.7	35.3	8.8	14.3	21.3	0.000 (0.0)	64
**PF (0.8)**	11.9 (11.5–12.3)	**0.317** **(14.7)**	5.5	38.4	6.1	10.8	17.1	0.000 (0.0)	45
**CBN (0.8)**	13.3 (12.8–13.8)	**0.762** **(14.7)**	4.3	26.0	5.8	11.5	19.4	0.000 (0.0)	56
**UI (0.4)**	8.6 (8.3–9.0)	**0.852** **(14.5)**	4.1	40.6	3.5	7.3	12.7	0.000 (0.0)	28
**CN_2_3 (0.6)**	17.9 (17.5–18.4)	**0.590** **(14.5)**	8.0	36.2	10.6	16.6	23.6	0.000 (0.0)	75

- **Normalized number of bigrams:** The normalized number of bigrams of the synthetic lists generated by the models is reported in Table 6. Statistical validation confirms that all evaluated models follow a Gaussian distribution, supported by high Bayes factors (BF_01_) and nonsignificant *p*-values. Despite this structural consistency, most unweighted methods fail to produce a meaningful number of relevant bigrams, with means consistently below 2.0 compared to the training average of 5.37. The CN_2_3 model is the only notable exception, generating a distribution that is statistically equivalent to the training data in terms of quantity. It achieves a mean of 5.46, a high Bayes factor (10.3), and a 97% overlap, indicating that it successfully replicates the aggregate volume of bigrams found in human lists. However, the low *R*^2^ score (0.26) for CN_2_3 reveals a critical limitation: while the quantity of bigrams is correct, the specific associations do not match human performance. This result is expected for unweighted models, as they treat all transitions from a node as equally probable. This inability to prioritize specific lexical links reinforces the conclusion that unweighted architectures cannot fully reflect the selective nature of human associative memory.

**Table 6:** Normalized number of bigrams of unweighted methods

Dataset (jump prob.)	Mean	Standard deviation	*p*-value -Gaussian-(BF_01_)	*p*-value -training-(BF_01_)	Overlap %	*R*^2^
**Training**	5.37	1.87	**0.173** **(10.8)**	NA	NA	NA
**Testing**	5.07	1.78	**0.987** **(6.8)**	**0.090** **(2.1)**	**93**	**0.86**
**FE (0.8)**	1.13	1.54	**0.627** **(14.7)**	0.000 (0.0)	21	−0.04
**NRW (0.8)**	1.11	1.48	**0.462** **(14.5)**	0.000 (0.0)	20	−0.09
**PF (0.8)**	1.54	1.54	**0.498** **(3.5)**	0.000 (0.0)	26	0.05
**CBN (0.8)**	1.77	1.59	**0.623** **(13.7)**	0.000 (0.0)	30	0.22
**UI (0.4)**	1.48	1.59	**0.604** **(13.0)**	0.000 (0.0)	26	−0.18
**CN_2_3 (0.6)**	**5.46**	**1.79**	**0.072** **(14.7)**	**0.051** **(10.3)**	**97**	0.26

### Assessment of weighted methods

- **Global likelihood:** The global likelihood measure for the weighted methods is reported in Table 7. The analysis of the weighted methods reveals a significant improvement in capturing the associative nuances of the data, with all models producing *gLL* distributions that fit a beta distribution according to *p*-values and Bayes factors. The evidence suggests that the training and testing data are statistically equivalent under the BIGRAM-IND, BIGRAM-CN (for both $\gamma$ values), and CN-INVITE models. While the *p*-values and Bayes factors for this equivalence are relatively weak, the distributional overlap exceeds 90% in all these cases. However, the requirement for generative quality reveals that only BIGRAM-CN ($\gamma =1.10$) and CN-INVITE produce synthetic lists with strong evidence of equivalence to the human training data, showing robust Bayes factors of 7.8 and 12.8, respectively. In contrast, the BIGRAM-FIXED and INVITE methods show clear signs of overfitting, characterized by high training verisimilitude that fails to generalize to test or synthetic data. Furthermore, while the BIGRAM-IND method maintains high overlap, its synthetic output is “overly likely” (2.87%) compared to the training baseline (2.25%), suggesting a bias toward overrepresenting established associations. Finally, when evaluating these models through the lens of parsimony, the BIGRAM-CN ($\gamma =1.10$) method appears superior. Despite the similar likelihoods achieved by CN-INVITE, its significantly higher BIC (25,899 vs. 17,840 for CN-BIGRAM) indicates that its increased structural complexity and higher number of parameters are not justified by the marginal gains in global likelihood.

**Table 7:** Global likelihood results of weighted methods

Dataset (param.)	Data	*gLL* % (95% CI)	BIC	*p*_value beta dist (BF_01_)	Alpha	Beta	Bottom-25%	Mode	Hi-25%	*p*_value train (BF_01_)	Overlap %
**BIGRAM-FIXED** **(JP = 0.4)**	Train	3.23 (3.17–3.29)	60,974	**0.996** **(14.3)**	16.9	495.4	2.29	3.12	4.08	NA	NA
	Test	1.83 (1.63–2.03)	18,255	**0.294** **(6.2)**	2.9	127.9	0.50	1.49	3.09	0.000 (0.0)	48
	Synt.	1.85 (1.80–1.91)	70,830	**0.286** **(14.5)**	8.9	446.0	1.08	1.75	2.52	0.000 (0.0)	34
**BIGRAM- IND**	Train	2.25 (2.14–2.37)	67,395	**0.864** **(13.7)**	3.1	110.0	0.70	1.85	3.78	NA	NA
	Test	1.91 (1.69–2.13)	18,046	**0.710** **(7.4)**	2.5	102.9	0.40	1.48	3.32	0.095 (0.8)	**91**
	Synt.	2.87 (2.77–2.97)	63,044	**0.827** **(13.0)**	6.2	195.0	1.45	2.62	4.10	0.000 (0.0)	80
**BIGRAM-CN** **(**${\boldsymbol{\gamma}}=1)$	Train	**2.30** **(2.20–2.40)**	67,015	**0.812** **(8.7)**	3.7	134.7	0.86	1.97	3.70	NA	NA
	Test	**2.01** **(1.81–2.21)**	17,842	**0.601** **(5.5)**	3.0	122.2	0.58	1.65	3.35	**0.176** **(1.3)**	**91**
	Synt.	2.11 (2.04–2.18)	68,540	**0.796** **(14.5)**	6.2	262.1	1.11	1.94	3.09	0.000 (0.0)	83
**INVITE**	Train	3.68 (3.58–3.79)	1,112,687	**0.943** **(14.1)**	8.4	207.3	2.12	3.48	5.04	NA	NA
	Test	1.74 (1.57–1.92)	914,861	**0.922** **(7.5)**	3.0	136.4	0.49	1.44	2.95	0.000 (0.0)	45
	Synt.	1.21 (1.15–1.28)	1,132,389	**0.002** **(7.7)**	2.3	146.4	0.20	0.89	2.14	0.000 (0.0)	29
**CN-INVITE**	Train	**2.28** **(2.18–2.40)**	76,576	**0.859** **(14.7)**	3.4	125.5	0.78	1.92	3.70	NA	NA
	Test	**1.99** **(1.80–2.19)**	25,899	**0.825** **(6.2)**	3.0	122.0	0.56	1.62	3.32	**0.084** **(1.0)**	**92**
	Synt.	**2.35** **(2.27–2.45)**	76,043	**0.237** **(11.6)**	4.7	173.4	1.06	2.09	3.60	**0.028** **(12.8)**	**92**
**BIGRAM-CN** **(**${\boldsymbol{\gamma}}=1.10)$	Train	**2.31** **(2.20–2.41)**	**66,957**	**0.897** **(5.4)**	3.5	125.4	0.76	1.95	3.70	NA	NA
	Test	**2.01** **(1.81–2.22)**	**17,840**	**0.579** **(7.6)**	2.9	116.4	0.56	1.63	3.39	**0.156** **(1.3)**	**91**
	Synt.	**2.34** **(2.26–2.43)**	**66,696**	**0.529** **(12.0)**	5.0	187.1	1.05	2.11	3.51	**0.042** **(7.8)**	**90**

- **Word frequency likelihood:** The word frequency likelihood measure is reported in Table 8. The results for the frequency likelihood of the weighted methods consistently follow a beta distribution, as confirmed by nonsignificant *p*-values and supportive Bayes factors. Only the BIGRAM-CN and CN-INVITE models produce *fLL* values with confidence intervals comparable to the human training baseline, whereas the INVITE method shows a strikingly low likelihood of 6.0%. This poor performance by INVITE suggests that a jump mechanism is necessary to properly organize information and replicate human clustering and switching behaviors. Although the distributional overlap remains high (around 89%) for most top methods, only BIGRAM-CN ($\gamma =1.10$) provides strong Bayesian evidence (BF_01_ = 10.0) and a nonsignificant *p*-value (0.210) to indicate that its synthetic lists are statistically equivalent to the training data. Additionally, it is noteworthy that the BIGRAM-IND model produces an inflated *fLL* of 26.5%, which likely indicates that it overrepresents high-frequency transitions and fails to capture the natural variability seen in the training set.

**Table 8:** Frequency likelihood results of weighted methods

Dataset (param.)	fLL % (95% CI)	*p*-value_beta (BF_01_)	Alpha	Beta	Low-25% %	Mode %	Hi-25% %	*p*-value_train (BF_01_)	Overlap %
**Training**	21.2 (20.5–21.9)	**0.088** **(13.9)**	5.7	19.7	11.7	20.1	30.2	NA	NA
**Testing**	19.6 (17.9–21.1)	**0.201** **(6.4)**	4.0	14.6	8.9	17.9	29.7	**0.386** **(4.0)**	**90**
**BIGRAM-FIXED** **(JP = 0.4)**	19.1 (18.5–19.7)	**0.291** **(13.0)**	6.3	25.7	10.9	17.7	26.6	0.000 (0.0)	**86**
**BIGRAM-IND**	26.5 (25.8–27.1)	**0.255** **(7.8)**	8.1	22.4	16.8	25.0	34.9	0.000 (0.0)	78
**BIGRAM-CN** **(**${\boldsymbol{\gamma}}=1)$	**20.7** **(20.2–21.2)**	**0.923** **(4.3)**	8.1	30.3	12.6	19.5	27.4	0.000 (0.0)	**89**
**INVITE**	6.0 (5.6–6.4)	**0.336** **(14.3)**	1.5	16.6	0.2	3.0	11.5	0.000 (0.0)	31
**CN-INVITE**	**20.0** **(19.4–20.5)**	**0.365** **(13.9)**	6.8	26.5	11.5	18.6	27.1	0.000 (0.0)	**89**
**BIGRAM-CN** **(**${\boldsymbol{\gamma}}=1.10)$	**21.8** **(21.3–22.3)**	**0.620** **(7.8)**	8.7	30.7	13.9	20.6	28.8	**0.210** **(10.0)**	**89**

- **Normalized number of bigrams:** The normalized number of bigrams in the synthetic lists generated by the models is reported in Table 9. The results for the number of relevant bigrams (nRB) confirm that weighted architectures are essential for capturing human-like associative patterns. All weighted models produce distributions that follow a Gaussian model, as evidenced by the nonsignificant *p*-values and supportive Bayes factors (BF_01_ > 6.8). These methods show a marked improvement over unweighted models: while unweighted methods struggled to exceed a mean of 2.0, the weighted models consistently generate between 3.06 and 5.58 bigrams, aligning closely with the human training average of 5.37. A key finding in this metric is the strong correlation between distributional overlap and lexical identity (*R*^2^). Unlike unweighted models, where a correct quantity of bigrams did not guarantee the correct associations, these models show that high overlap (above 85%) is tied to high *R*^2^ scores (between 0.80 and 0.96). This confirms that the models are generating the right specific links in the appropriate proportions. Statistical equivalence to the training data is most prominent in the CN-INVITE and BIGRAM-CN ($\gamma =1.10$) models. The CN-INVITE method achieves the highest overlap (97%) and the strongest statistical evidence for equivalence, with a *p*-value of 0.530 and a Bayes factor (BF_01_) of 10.1. Similarly, the BIGRAM-CN $\gamma =1.10$) model shows the highest lexical precision, with an *R*^2^ of 0.96 and a high overlap (94%), supported by a BF_01_ of 2.6. Other models, such as BIGRAM-FIXED or INVITE, fail to reach these levels of equivalence, showing significantly lower overlap and failing the statistical tests against the training baseline.

**Table 9:** Normalized relevant bigrams results of weighted methods

Dataset (jump prob.)	Mean	Standard deviation	*p*-value -Gaussian- (BF_01_)	*p*-value -training- (BF_01_)	Overlap %	*R*^2^
**Training**	5.37	1.87	**0.173** **(10.8)**	NA	NA	NA
**Testing**	5.07	1.78	**0.987** **(6.8)**	**0.090** **(2.1)**	**93**	**0.86**
**BIGRAM-FIXED** **(JP = 0.4)**	3.06	1.71	**0.320** **(8.8)**	0.000 (0.0)	52	0.60
**BIGRAM-IND**	4.72	1.75	**0.687** **(12.8)**	0.000 (0.0)	85	**0.91**
**BIGRAM-CN** **(**${\boldsymbol{\gamma}}=1)$	**5.09**	**1.75**	**0.213** **(6.8)**	0.000 (0.7)	**93**	**0.94**
**INVITE**	3.35	2.01	**0.214** **(10.3)**	0.000 (0.0)	60	**0.80**
**CN-INVITE**	**5.47**	**1.87**	**0.451** **(12.5)**	**0.530** **(10.1)**	**97**	**0.90**
**BIGRAM-CN** **(**${\boldsymbol{\gamma}}=1.10)$	**5.58**	**1.75**	**0.765** **(14.1)**	**0.015** **(2.6)**	**94**	**0.96**

### Empirical validation of jump behavior

To ensure our model's compatibility with traditional frameworks for semantic switching (e.g., Troyer et al., 1997), we validated whether our predicted jumps aligned with transitions between semantic categories. In the absence of a standardized taxonomy for the clothing category in Spanish, we generated a classification using a large language model (LLM), later supervised by the research team. Each word was assigned to one or more semantic categories (e.g., anatomical location, context of use, or material). A transition was identified as a “jump” if the two words shared no common categories. While this definition is conservative and tends to reduce the absolute number of identified switches, it establishes a robust and reasonable framework for evaluating the predictive capacity of our model's indicators.

In this context, we analyzed two local topological metrics: node degree and semantic cohesion. Node degree was defined as the total number of edges connected to a node. Semantic cohesion was calculated as the probability of returning to a node within three steps, providing a weighted measure analogous to the clustering coefficient. This measure was computed using an equivalent transition matrix where the jump node was reabsorbed, preventing the transition through a jump from being counted as one of the three steps.

**Fig. 7:**
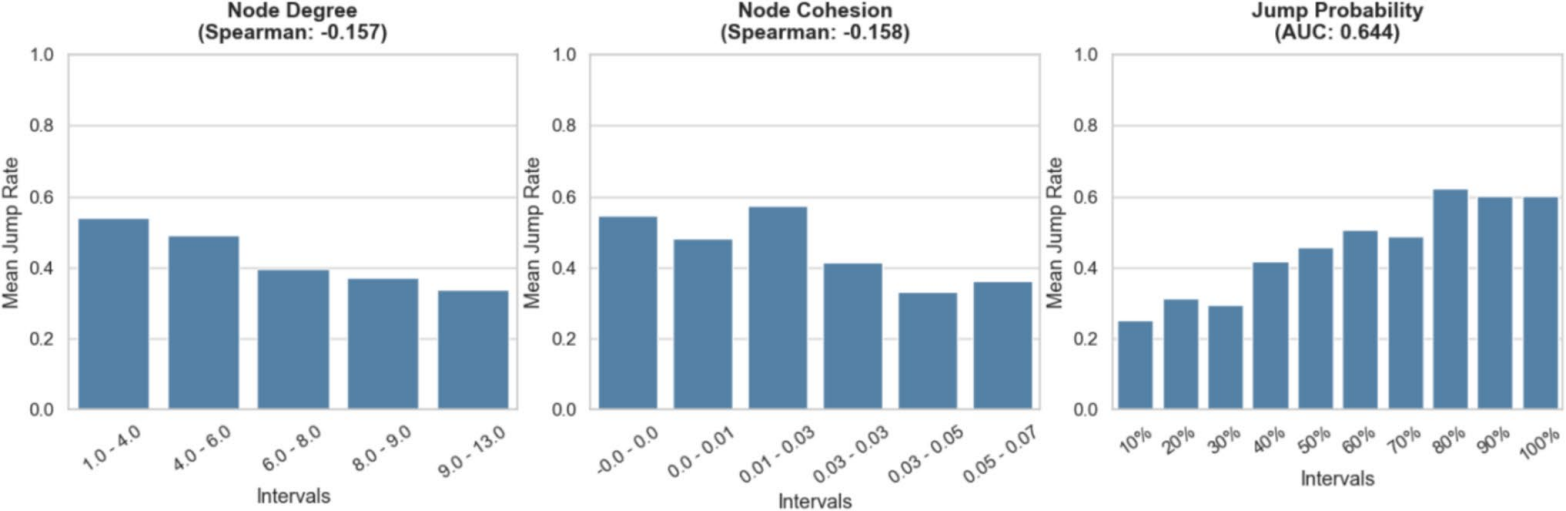
Mean jump rate from nodes by node degree, node cohesion, and jump probability

The results confirm that transitions to other communities (jumps) occur more frequently after visiting nodes with lower degree and lower semantic cohesion, as shown in Fig. 7. Specifically, we found a significant, negative rank correlation between jump occurrence and both node degree ($\rho$ = −0.157, *p* < 0.001) and semantic cohesion $\rho$ = −0.158, *p* < 0.001).

**Fig. 8:**
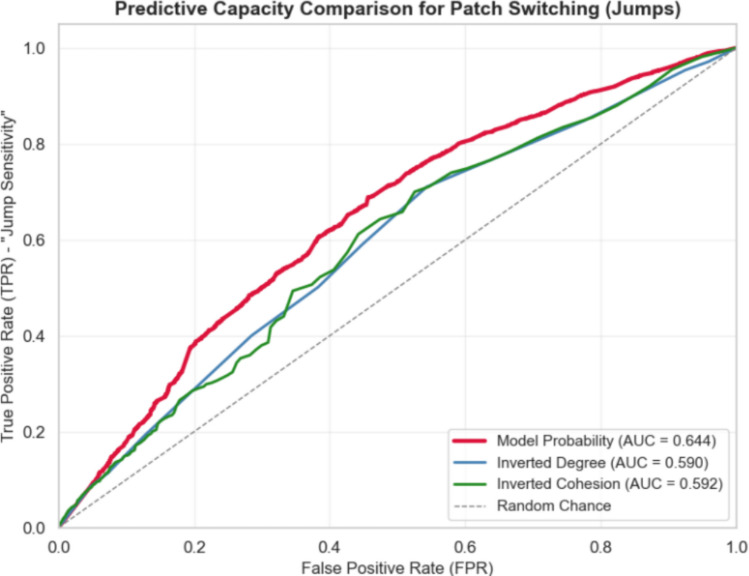
Receiver operating characteristic (ROC) curves for the prediction of patch switching (jumps). The plot compares the predictive performance of the proposed model-derived jump probability against baseline local topological metrics: inverted node degree and inverted semantic cohesion

Furthermore, we used this check to contextualize the predictive power of our proposed node-specific jump probabilities. We conducted a receiver operating characteristic (ROC) curve analysis to predict jumps; while inverted degree and inverted cohesion achieved area under the curve (AUC) values of 0.590 and 0.592, respectively, our calculated jump probability achieved a notably higher AUC of 0.644 (see Fig. 8). To ensure statistical rigor, we applied DeLong's test, which confirmed that our jump probability metric is a significantly better predictor than both local topological metrics (*Z* > 10.1, *p* < 0.001 in both comparisons). This demonstrates that while jumps are indeed more likely after low-degree/low-cohesion nodes, our proposed probability metric captures additional, complex semantic dynamics of the switching process.

**Fig. 9:**
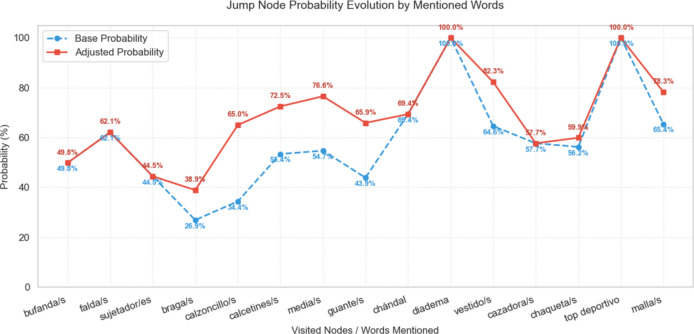
Evolution of jump probability as words are mentioned. Base probability is adjusted as words from the same cluster are mentioned, increasing the actual probability of switching patches

The jump probability is dynamic and is not determined solely by the node’s properties, as it evolves as the current cluster is exhausted. To illustrate this phenomenon, Fig. 9 displays the evolution of the jump probability across a sequence of words. The figure includes the base probability (direct transition to the jump node) and the adjusted probability (transition to the jump node through previously visited nodes). Significant discrepancies emerge while navigating within a cluster and vanish upon switching to a new cluster.

## Discussion

As outlined in the introduction, the semantic fluency task—which involves linguistic processing, global attentional activation, executive control, and strategic search mechanisms—serves as an indicator of semantic memory access. Consequently, its outcomes have been widely used to develop cognitive models of semantic retrieval. Strategic search behavior reflects dynamic processes characterized by transitions between lexical items, typically conceptualized as jumps across network nodes or movements within spatial representations.

Advancing more sophisticated models of fluency task performance has direct implications for diagnosing differential linguistic behavior across specific populations. This constitutes the primary long-term objective of our research, in line with recent proposals by Neergaard et al. (2025) or Fatima et al. (2021). Accordingly, we adopt random-walk approaches (Zemla & Austerweil, 2017) as a foundational framework while enhancing the discriminative power of existing models through targeted modifications. These refinements primarily address overfitting and provide a coherent account of switching and clustering mechanisms. As a result, we identify metrics that more precisely capture the underlying cognitive operations and facilitate the detection of dimensions along which particular groups diverge most strongly. In what follows, we summarize the main contributions of BIGRAM-CN and the comparison with other previous models.

### Representation and navigation method

The semantic network is represented through a TPM navigated by a CRW. While this approach is not new, the method introduces a key innovation that enhances modeling power, visualization, and analytical flexibility.

In Jun et al. (2015), the TPM permits all transitions between nodes, and therefore does not require a jump mechanism. In contrast, the TPM proposed in Zemla and Austerweil (2018) restricts transitions to binary values (0 or 1), making many transitions impossible and assigning zero likelihood to any list containing such transitions. Such limitation is addressed by introducing a reset mechanism (jump) with a fixed probability.

Rather than assigning a uniform jump probability to all nodes—a simplification that does not reflect the structure of semantic memory—we propose the introduction of a fictional node that mediates global jumps. This “jump node” is integrated into the TPM and allows for flexible modeling of mental resets and long-range transitions.

This design offers several advantages including the individual tuning of jump probabilities for each node which, in turn, act as a measure of cluster depletion. It disentangles genuine semantic links from those arising due to mental resets and it maintains a Markovian structure which allows us to apply tools like stationary distribution and Markov stability.

- **Metrics and benchmark:** Although maximizing likelihood is a fundamental technique for model fitting, it is not commonly applied in models of verbal fluency tasks. The definition and use of likelihood proposed in Jun et al. (2015) and Zemla and Austerweil (2018) is extremely valuable and serves as the foundation of this work. However, there are certain phenomena that are not fully captured by likelihood alone, or that, when likelihood is analyzed in greater depth, reveal underlying causes for a given fit.

The first of these is the phenomenon of overfitting. A model should not only fit the training data well, but it must also generalize to unseen data of the same nature. We assess this issue by comparing the likelihood of training lists with that of test lists. A KS test, coupled with Bayes factor, is used to evaluate whether the likelihoods for training and test data can be considered as coming from the same underlying distribution. Models such as NRW, PF, UI, BIGRAM-FIXED, and INVITE show significant differences between training and test data, indicating clear signs of overfitting—even when some of them include mechanisms designed to prevent it.

Another way to assess overfitting is by measuring the overlap between the distributions fitted to test data and to synthetic lists generated and the distribution fitted to training data. This approach checks not only whether the model behaves consistently across datasets, but also whether the data it generates follow the same statistical properties as the original data. The INVITE-CN and BIGRAM-CN models are the only ones that pass the test on this front and achieve the highest overlap scores.

There are situations in which overfitting is not entirely undesirable. Specifically, the CN models include a parameter that sets a minimum number of co-occurrences required for a transition to be considered in the analysis. This parameter, for instance, allows the exclusion of transitions that occur only twice across 500 lists, even if statistical analysis indicates that such co-occurrence is significant. There are valid arguments both for retaining and for discarding such transitions. On one hand, statistical analysis shows the transition to be relevant, even if it is infrequent. On the other hand, if it occurs in less than 1% of the lists, it is unlikely to appear in future data, and its inclusion may increase the likelihood discrepancy between datasets. We argue that the researcher’s judgment and the intended function of the model should guide this decision. In our case, we opted to maximize likelihood through a fivefold cross-validation process within the training data.

The remaining metrics were defined considering that the variables affecting the likelihood of a list are its length, the specific words it contains, and the order in which the words are mentioned. Therefore, these measures allow us both to assess whether an individual or a population produces a list that aligns with a given model, and to identify the specific characteristics of their list in relation to the model.

Each of the three metrics was used to model both training and test data in order to verify whether the measure generalizes to unseen data. In addition, scores were computed for lists synthesized by each model to assess whether they were statistically similar to the original ones. This analysis helps identify how a random model generates extremely improbable words, whereas a model based solely on word frequency yields lists with high frequency-based likelihood but virtually no occurrence of relevant bigrams.

The applications of these measures are broad, spanning linguistics, psychology, and neuropsychology. First, they enable the comparison of models in order to identify the most accurate way to represent the list-generation process of a given population. Second, once the model’s quality is established, these measures allow for the identification of differences between models fitted to different groups (e.g., male/female, children/adults, native/non-native speakers, clinical/nonclinical populations), across various semantic categories (natural categories, ad hoc categories, schemas, etc.), and along different dimensions (e.g., overall likelihood, list length, word frequency, and associative structure).

Furthermore, having multiple models for different word categories may support the diagnosis of individuals who either are in the process of learning a language or exhibit cognitive impairments, by providing scores (e.g., in percentile form) for each category and each metric.

### Performance of the proposed technique

Several techniques for constructing TPMs have been analyzed. The simplest approaches use all or part of the lists to identify possible transitions in a binary fashion (e.g., FE, NRW). Others rely on more convoluted methods to determine relevant associations (e.g., PF, CN, CBN, UI), although these also neglect the possibility of weighting relationships. Finally, methods such as BIGRAM and INVITE allow for weighted links, enabling transition probabilities from a node to follow a nonuniform distribution across connected nodes. However, these approaches tend to result in significant overfitting.

Models that constrain the number of parameters, while still allowing weighted connections (e.g., BIGRAM-IND, BIGRAM-CN, INVITE-CN), are the ones that avoid overfitting while maintaining nearly equivalent performance in terms of overall likelihood. A direct comparison between BIGRAM-IND and BIGRAM-CN reveals that the latter performs slightly better across all metrics. The frequency likelihood metric shows that only the BIGRAM-CN model with γ =1.10 generates lists with a word frequency distribution truly comparable to that of the original data. The bigram metric shows that both BIGRAM-CN and INVITE-CN produce a distribution of relevant bigrams equivalent to that of the training data.

We can therefore conclude that the BIGRAM-CN model generation technique provides the best overall fit across all metrics and is particularly suitable for synthesizing lists similar to those observed in training.

## Conclusions

The SFT is a well-established procedure used in linguistics to identify the underlying semantic network. It is also a common practice in psychology to use SFT to assess a patient’s lexical access and semantic memory. Several techniques have been proposed in the literature to obtain a model that can mimic the process and assess the associative retrieval of words, but there is a lack of consensus regarding the quality and accuracy of such models. This paper proposes a modeling technique based on the common practice of maximizing likelihood. The model identifies the SFT as a censored random walk over a network that includes local edges that represent the associative retrieval but also introduces a pseudo-node that makes it possible to reach any node at any time to model mental resets. Additionally, several types of pseudo-nodes can be included to model partial resets. This dichotomy between local and global links allows us to analyze the clustering of words that typically arise in these processes while also modeling the switching between clusters or even idiosyncratic words.

While likelihood is the main quality measure of the model, three additional metrics are defined. These metrics aim to assess the quantity of words, frequency of words, and association among words. They can be used to assess the quality of a model but also provide a useful evaluation for individual or group lists, as they act as a benchmark to compare an individual or a population to a set standard, providing a thorough assessment of the SFT more relevant than simply counting words.

The proposed model was tested against many of the models reported in the recent literature and was found to be more accurate than unweighted models, because the uniform distribution does not accurately represent the probability of transitioning between nodes, even if only statistically relevant edges are included. Our proposal is able to generalize more accurately than previous weighted techniques because these models appear to have an excessive number of parameters and are prone to overfitting.

Overall, the model opens a wide range of possibilities in the field of linguistics, psychology, and clinical neuropsychology. If fitted to the proper data, the model and its metrics can help detect differences between populations (male/female, regions of origin, language proficiency…) that had not been previously detected. It can also be used to better understand the differences in memory retrieval between semantic categories, given that the dynamic Markovian approach provides a more accurate representation than the previous static network theory. It is also an inexpensive method that can be applied to existing databases and expands their diagnostic capabilities of cognitive degenerative processes like Alzheimer’s or dementia.

Moreover, although not the central aim of the present study, our contributions also inform ongoing debates on semantic memory search models by highlighting points of convergence between optimal foraging and random-walk formulations, tempering some of the criticisms among them. Our review and analysis of the computational implementations reveal that both perspectives are, in their current formulations, mathematically equivalent. The sequential contributions of Hills, Avery, and Zhang have refined the foraging model into a structure that is extraordinarily analogous to a CRW.

This structural equivalence becomes evident when examining the mechanical similarities between the models. On the one hand, the local cue proposed by Hills and later used in Avery and Jones (2018) assigns a transition probability between an item *X*_*n*_ and *X*_*n+*1_ based on semantic similarity and frequency, and it operates exactly like the “local” submatrix of a Markov process that incorporates a jump node. Within this framework, the global cue effectively functions as the probability distribution that governs landing on a new node after executing such a jump.

As acknowledged by Zhang and Jones (2022), the early cue switch model presented a methodological limitation by evaluating the transition between cues postdictively; that is, they assigned a jump probability of 1 assuming a posteriori knowledge that the participant had switched clusters. However, the introduction of the predictive mechanism by Zhang and Jones (2022) rectifies this irregularity by proposing a dynamic estimation of the jump probability. This contribution essentially provides the final vector necessary to completely define the random walk's TPM.

There are, nevertheless, differences between the two approaches, although they appear to be of a rather formal nature rather than conceptual. First, the jump probability in the Hills–Zhang model is dynamic and recalculated after each visit, distancing it from a pure Markov process. Second, the models differ in their approach to censoring previously produced items. While Abbott's CRW maintains the underlying transition probabilities intact before removing the nodes from the candidate list, the joint model of Hills, Avery, and Zhang directly alters the topological matrix, forcing the link weights to already visited nodes to zero. Despite these divergences, which should be more profoundly studied, the fundamental underlying dynamics appear to be equivalent, although these approaches will need to be validated through further comparative analyses and networks from data of different nature.

Moreover, our progress in semantic network modeling may contribute to discussions on how behaviorally derived models can be integrated with corpus-based approaches, as suggested by Kumar et al. (2022). Explaining why our model achieves greater likelihood and stronger generalization capacity may also clarify the limitations of text-based spatial representations as proxies for semantic memory structure.

To conclude, this work presents a unified and flexible framework for modeling semantic fluency that not only improves model fit and generalization capacity compared to previous approaches but also introduces new metrics for a richer and more nuanced evaluation of semantic performance. These contributions lay the groundwork for future empirical and contrastive research (such as evaluating the model on embeddings-based or associative-norms-based networks, as well as carrying out other psycholinguistic tasks as external validation) and reinforce the potential of the SFT as a sensitive and robust research tool across diverse domains.

## Data Availability

The datasets and source used in this study are openly available at https://github.com/mlopezgUMH/VFTGraph.

## Appendix 1

The probability of two words occurring at a distance *d* is calculated as follows:

The probability of a word *w*_*i*_ to appear on a list can be expressed as

13 $${\mathbb{P}}_{{w}_{i}}=\frac{{f}_{{w}_{i}}}{N},$$

where *f*_*wi*_ is the number of lists in which *w*_*i*_ appears and N is the total number of lists.

Considering that the co-occurrence of two words on the same list is independent then the probability of co-occurrence is:

14 $${\mathbb{P}}_{{w}_{i},{w}_{j}=}{\mathbb{P}}_{{w}_{i}}\cdot {\mathbb{P}}_{{w}_{j}}$$

The probability of two words on the same list appearing at a distance *d* is expressed as:

15 $${\mathbb{P}}_{{w}_{i}{w}_{j}}^{d}=2\frac{\overline{\lambda}-d}{\left[\begin{array}{c}\overline{\lambda}\\ 2\end{array}\right]}=2\frac{\overline{\lambda}-d}{\overline{\lambda }\left(\overline{\lambda }-1\right)},1\le d<\overline{\lambda},$$

where $\overline{\lambda }$ is the mean length of the lists, *2·(*$\overline{\lambda}$*-d)* is the number of positions in which the words are at distance *d* and $\overline{\lambda }$*·(*$\overline{\lambda }$*-1)* is the total number of positions that the words can occupy in the list.

The probability of the words appearing at distance *d* or closer is:

16 $${\mathbb{P}}_{{w}_{i}{w}_{j}}^{\le d}=2\sum\nolimits_{i=1}^{d}\frac{\overline{\lambda }-i}{\left[\begin{array}{c}\overline{\lambda}\\ 2\end{array}\right]}=\frac{2}{\overline{\lambda }(\overline{\lambda }-1)}\left(d\cdot \overline{\lambda}-\frac{d(d+1)}{2}\right),1\le d<\overline{\lambda }$$

The final probability of two words appearing on the same list at a distance d or shorter is therefore:

17 $${\mathbb{P}}_{{w}_{i}{w}_{j}}^{link}={\mathbb{P}}_{{w}_{i},{w}_{j}}\cdot {\mathbb{P}}_{{w}_{i}{w}_{j}}^{\le d}=\frac{{f}_{{w}_{i}}{f}_{{w}_{j}}}{{N}^{2}}\cdot \frac{2}{\overline{\lambda}(\overline{\lambda }-1)}\left(d\cdot \overline{\lambda }-\frac{d(d+1)}{2}\right),1\le d<\overline{\lambda}$$

The Clopper and Pearson method (Clopper & Pearson, 1934) is used to determine the confidence interval for the binomial distribution based on the number of attempts and success observed. If the probability calculated is lower than the lower bound, then the hypothesis that the distances between words is due to chance can be rejected and the link is validated. The number of co-occurrences must be sufficient for the method to provide a narrow confidence interval. In Goñi et al. (2011), this threshold is set to one, excluding all words that do not co-occur more than once. Additionally, they also set the distance limit to 2, arguing there is not semantic relation beyond that distance (Troyer et al., 1998).

## Code availability

The datasets and source code used in this study are openly available at https://github.com/mlopezgUMH/VFTGraph.
